# Metformin as antiviral therapy protects hyperglycemic and diabetic patients

**DOI:** 10.1128/mbio.00634-25

**Published:** 2025-05-20

**Authors:** Xi Wang, Xiaojie Zheng, Honghan Ge, Ning Cui, Ling Lin, Ming Yue, Chuanlong Zhu, Qi Zhou, Peixin Song, Xing Shang, Rui Wang, Zhen Wang, Zhiyou Wang, Yunfa Zhang, Xiaohong Yin, Linsheng Yang, Hong Su, Hao Li, Wei Liu

**Affiliations:** 1 School of Public Health, Anhui Medical University 569061 , Hefei, Anhui, China; 2 State Key Laboratory of Pathogen and Biosecurity, Academy of Military Medical Sciences 602528, Beijing, China; 3 School of Public Health, Shandong First Medical University and Shandong Academy of Medical Sciences 518873, Jinan, Shandong, China; 4 The 154th Hospital, China RongTong Medical Healthcare Group Co.Ltd, Xinyang, Henan, China; 5 Yantai Qishan Hospital, Yantai, Shandong, China; 6 The First Affiliated Hospital of Nanjing Medical University, Nanjing, Jiangsu, China; 7 Shandong Provincial Public Health Clinical Center 442536 , Jinan, Shandong, China; 8 Nanjing Drum Tower Hospital 66506, Nanjing, Jiangsu, China; 9 College of Veterinary Medicine, Yangzhou University 614704, Yangzhou, Jiangsu, China; Johns Hopkins University, Baltimore, Maryland, USA

**Keywords:** severe fever with thrombocytopenia syndrome virus, hyperglycemia, diabetes, metformin, antiviral therapy

## Abstract

Viral infections disrupt glucose metabolism; however, their impact on disease prognosis in highly pathogenic viruses remains largely unknown. There is an additional need to investigate the antiviral mechanisms of glucose-lowering therapeutics. Here, our multicenter clinical study shows that hyperglycemia and pre-existing diabetes are independent risk factors for mortality in patients infected with severe fever with thrombocytopenia syndrome virus (SFTSV), an emerging and highly pathogenic bunyavirus. SFTSV infection triggers gluconeogenesis, which, in turn, inhibits AMPK activity and subsequent interferon I (IFN-I) responses, thereby facilitating viral replication. *In vitro* and animal studies further reveal that metformin inhibits SFTSV replication by suppressing autophagy through the AMPK-mTOR pathway, contributing to protection against lethal SFTSV infection in mice. Importantly, our large cohort study demonstrates that metformin reduces viremia and SFTSV-related mortality in patients with hyperglycemia or pre-existing diabetes, contrasting with the disadvantageous effect of insulin. These findings highlight the promising therapeutic potential of metformin in treating viral infections, particularly among individuals with hyperglycemia or diabetes.

**IMPORTANCE:**

Severe fever with thrombocytopenia syndrome virus (SFTSV), an emerging tick-borne bunyavirus, causes severe hemorrhagic fever with a high mortality rate. Previous studies have shown metabolic disturbances, particularly hyperglycemia, in SFTSV-infected individuals. However, the mechanism underlying this metabolic derangement remains unclear, and further investigation is needed to determine whether glucose-lowering therapeutics could be beneficial for SFTSV-infected patients. In this study, our multicenter clinical data show that hyperglycemia and pre-existing diabetes are independent risk factors for mortality in patients with SFTSV infection. Furthermore, we observed that SFTSV infection triggers gluconeogenesis, which promotes viral replication through the regulation of the AMPK-IFN-I signaling pathway. Notably, metformin significantly reduces viremia and SFTSV-related mortality in patients with hyperglycemia or pre-existing diabetes, attributed to its inhibitory effect on autophagy through the AMPK-mTOR pathway. Therefore, our study uncovers the interaction between SFTSV infection and glucose metabolic disorder and highlights the promising therapeutic potential of metformin for treating SFTSV infection.

## INTRODUCTION

The cross-talk between viral infections and host metabolism, such as glucose, fatty, and protein metabolism, is crucial for determining the prognosis of infection. Hosts undergo intricate metabolic adaptations to enhance immune function and promote tissue tolerance during viral infections. Meanwhile, viruses can dramatically alter host cell metabolism to create optimal environments for viral replication and dissemination. Notably, viruses can exploit glucose metabolism processes like glycolysis and gluconeogenesis, which maintain plasma glucose levels within a tight range (1, 2), to increase available energy and support viral reproduction. For instance, severe acute respiratory syndrome coronavirus 2 (SARS-CoV-2) induces mitochondrial dysfunction while Zika virus suppresses AMPK signaling to augment glycolysis within host cells (3, 4). Conversely, certain viruses induce hyperglycemia by targeting hepatocytes to trigger gluconeogenesis or infecting β-cells to impair insulin secretion (5–8). Although some pathways exploited by different classes of viruses have been studied in relation to replication and pathogenesis modulation, the underlying mechanisms are still not fully understood. It is noteworthy that the impact of virus-induced dysregulation of glucose metabolism on the prognosis of viral infections remains largely unexplored for highly pathogenic viruses.

Severe fever with thrombocytopenia syndrome virus (SFTSV), now renamed as *Bandavirus dabieense* in the family Phenuiviridae within the class Bunyaviricetes, is one of the most lethal viruses identified in the past decade. Infection with SFTSV frequently leads to severe or critical consequences, including hemorrhage, encephalitis, and multiple organ failure, with mortality rates ranging from 12% to 50% among hospitalized patients (9, 10). Since its discovery in China in 2009, SFTSV has emerged as a pandemic concern in Asia (11, 12). Previous studies have indicated metabolic disturbances, particularly hyperglycemia, in individuals infected with SFTSV (13). However, the underlying mechanism remained unclear, and further investigation is needed to determine whether there is a glucose-lowering therapeutic effect that could benefit SFTS patients.

In this multicenter study conducted across various provinces in China, we observed an increased risk of fatality among hyperglycemic or diabetic individuals infected with SFTSV and demonstrated the effectiveness of metformin against SFTSV infection. Furthermore, we discovered that SFTSV infection induces hyperglycemia through activation of the gluconeogenesis pathway while inhibiting AMPK activity and subsequent IFN-I response to facilitate viral replication. In addition, both *in vitro* and animal studies demonstrated that metformin suppresses SFTSV replication, which was associated with its inhibition of autophagy via the AMPK-mTOR pathway. These results support our clinical findings and highlight metformin’s therapeutic potential for treating SFTS.

## RESULTS

### SFTSV infection induces hyperglycemia that leads to poor prognosis in patients without pre-existing diabetes

**Fig 1:**
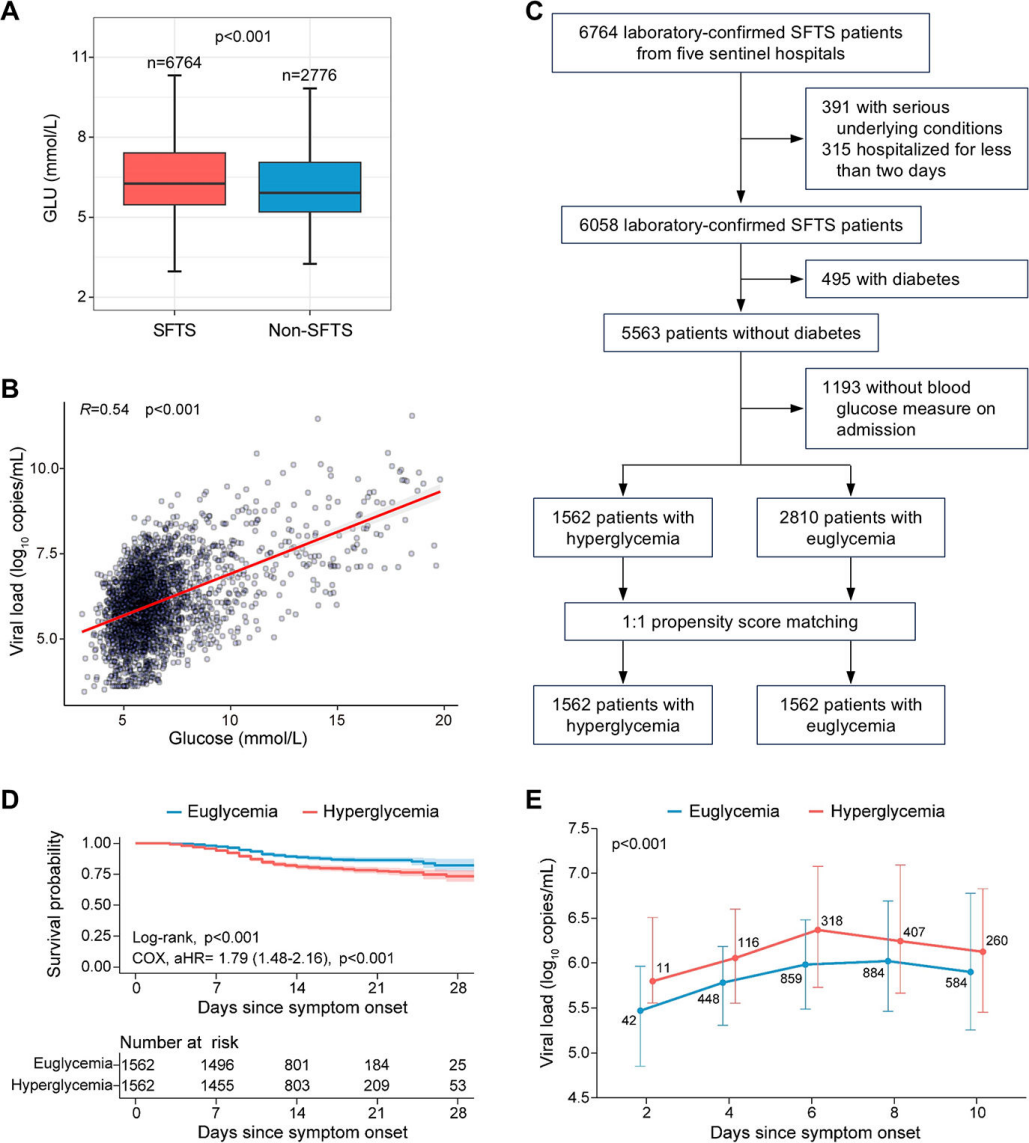
SFTSV infection induces hyperglycemia that leads to poor prognosis in individuals without underlying diabetes. (**A**) Comparison of blood glucose (GLU) levels at admission between SFTS and non-SFTS patients. The *P*-value was calculated by the Wilcoxon rank-sum test. (**B**) Pearson correlation coefficient between blood glucose levels and SFTSV viral loads in SFTS patients. (**C**) Flowchart of the recruitment and grouping process for SFTS patients. A total of 6,764 patients admitted into five sentinel hospitals for SFTS were included in the retrospective clinical investigation and divided into hyperglycemia and euglycemia groups using propensity score matching. (**D**) Analysis of hyperglycemia on the survival probability of SFTS patients. The Kaplan-Meier method was used to analyze time-to-event data. Adjusted HR and 95% CI were conducted by a multivariable COX regression with adjustment for age, sex, onset-to-admission interval, and pre-existing comorbidities. (**E**) Dynamic profiles of SFTSV viral loads between the hyperglycemia and euglycemia groups. Data were presented as median and interquartile range. A GEE model was performed to consider the effect of age, sex, and onset-to-admission interval. The number of SFTS patients included for analysis at each time point was added to the line graphs.

To investigate the effect of SFTSV infection on glucose metabolism, we conducted a clinical study involving 6,764 patients with laboratory-confirmed SFTSV infection and 2,776 febrile patients without SFTSV infection from five sentinel hospitals in China between 2011 and 2023. After excluding individuals with underlying diabetes, our analysis revealed that upon admission, patients with SFTSV infection exhibited significantly elevated blood glucose levels compared to the non-infected group (6.4 mmol/L vs. 6.0 mmol/L, *P* < 0.001; Fig. 1A). Notably, a positive correlation was observed between serum viral loads and blood glucose concentrations in patients with SFTS (*R* = 0.54, *P* < 0.001; Fig. 1B). These results suggest that SFTSV infection may disrupt glucose homeostasis, leading to hyperglycemia.

To further explore the consequences of hyperglycemia on disease progression, we employed propensity score matching to stratify SFTS patients into hyperglycemia group and euglycemia group (*n* = 1,562 for each group; Fig. 1C). Both groups were comparable in terms of demographic characteristics such as age and gender, as well as delay from symptom onset to hospital admission (Table S1). Survival analysis revealed a significantly higher fatality rate among patients with hyperglycemia compared to their euglycemic counterparts (18.6% vs. 10.9%, *P* < 0.001, log-rank test; Fig. 1D). This difference in fatality rates remained significant even after adjusting for confounding factors using multivariable COX regression analysis, which yielded a significant hazard ratio (HR) of 1.79 (95% CI, 1.48–2.16, *P* < 0.001; Fig. 1D). Moreover, the viral loads remained consistently higher throughout hospitalization for those experiencing hyperglycemia when compared to the euglycemic group (*P* < 0.001, generalized estimating equation [GEE] model; Fig. 1E). In addition, the hyperglycemic group exhibited more severe clinical manifestations, characterized by increased frequencies of respiratory and neurological symptoms (Table S1). When conducting sex-stratified analysis, the associations of hyperglycemia with increased fatality rates and enhanced viremia remain statistically significant in both male and female patients (Fig. S1). Collectively, these findings underscore the significance of hyperglycemia in association with SFTSV infection as a crucial risk factor that potentially augments viral replication and contributes to an unfavorable prognosis.

### SFTSV infection triggers gluconeogenesis to facilitate viral replication

**Fig 2:**
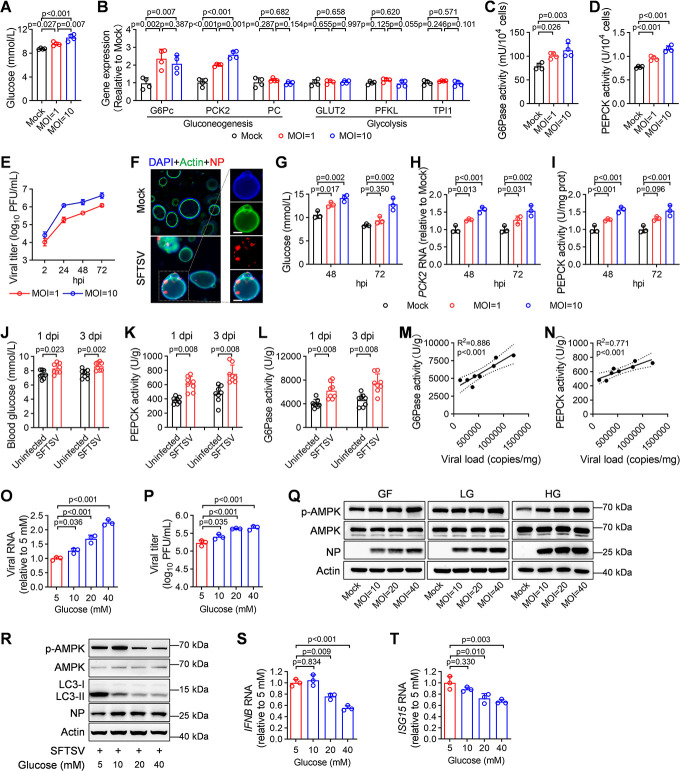
SFTSV infection triggers gluconeogenesis to facilitate viral replication. (**A**) Glucose level was measured in the supernatant of mock- and SFTSV-infected Huh7 cells at 48 hours post-infection (hpi); *n* = 4 biologically independent samples. (**B**) The mRNA levels of gluconeogenesis (*G6Pc*, *PCK2*, and *PC*) and glycolysis genes (*GLUT2*, *PFKL,* and *TPI1*) were measured in mock-infected and SFTSV-infected Huh7 cells at 48 hpi; *n* = 4 biologically independent samples. (**C, D**) The levels of G6pase (**C**) and PEPCK activity (**D**) were measured in mock- and SFTSV-infected Huh7 cells at 48 hpi; *n* = 4 biologically independent samples. (**E**) Viral titers were measured in the supernatant of primary human hepatocytes (PHHs)-derived liver organoids infected with SFTSV (MOI = 10) at 2, 24, 48, and 72 hpi; *n* = 3 biologically independent samples. (**F**) Immunofluorescence analysis of mock- and SFTSV-infected PHHs-derived liver organoids at 72 hpi. The enlarged images on the right are from SFTSV-infected PHHs-derived liver organoids. Scale bars, 100 µm. (**G–I**) The supernatant glucose levels (**G**), *PCK2* mRNA levels (**H**), and PEPCK activity (**I**) were measured in mock- and SFTSV-infected liver organoids at 48 and 72 hpi; *n* = 3 biologically independent samples. (**J–L**) The levels of blood glucose (**J**), PEPCK activity (**K**), and G6Pase activity (**L**) in liver from uninfected or SFTSV-infected mice at 1 and 3 days post-infection (dpi) (*n* = 8 per group). (**M, N**) Pearson correlation coefficient between viral loads and G6Pase activity (**M**) or PEPCK activity (**N**) in liver (*n* = 8). Dotted lines indicate the 95% confidence interval. (**O, P**) Intracellular SFTSV RNA level (**O**) and supernatant viral titers (**P**) were measured in SFTSV-infected Huh7 cells (MOI = 0.1) cultured with DMEM containing indicated glucose concentrations at 24 hpi; *n* = 3 biologically independent samples. (**Q**) Whole-cell extracts (WCEs) from SFTSV-infected Huh7 cells cultured in glucose-free (0 mM glucose, GF), low-glucose (5 mM glucose, LG), or high-glucose (20 mM glucose, HG). DMEM were analyzed by immunoblotting analysis using the indicated antibodies at 24 hpi. (**R**) WCEs from SFTSV-infected Huh7 cells (MOI = 1) cultured with DMEM containing indicated glucose concentrations were analyzed by immunoblotting analysis using the indicated antibodies at 24 hpi. (**S, T**) The mRNA levels of *IFNB* (**S**) and *ISG15* (**T**) were measured in SFTSV-infected Huh7 (MOI = 0.1) cultured with DMEM containing indicated glucose concentrations at 24 hpi; *n* = 3 biologically independent samples. Data were presented as mean ± s.d. The two-sided *P* values were examined using Student’s *t* test (**J**) or Wilcoxon test (**K, L**) for comparison of variables between two groups or one-way ANOVA followed by Tukey’s multiple comparisons test for comparison of continuous variables among multiple groups (**A–D, G–I, O, P, S, T**). The presented images are representative of three independent experiments in immunoblotting analysis (**F, Q, R**).

Given the pivotal role of the liver in regulating glucose homeostasis (2), we investigated the impact of SFTSV infection on glucose production in two human hepatic cell lines, Huh7 and HepG2, both known to be susceptible to SFTSV (14). Our analysis revealed that SFTSV infection induced an increase in glucose levels in the supernatant of both Huh7 and HepG2 cells, which was dependent on the multiplicity of infection (MOI) (Fig. 2A; Fig. S2A). Furthermore, gene expression analysis showed a significant upregulation of key gluconeogenic enzymes, including glucose-6-phosphatase catalytic (*G6Pc1*) and phosphoenolpyruvate carboxykinase (*PCK2*), in SFTSV-infected Huh7 cells at 48 hours post-infection (hpi) compared to mock controls (Fig. 2B). A consistent increase in *G6Pc1* expression was observed in SFTSV-infected HepG2 cells (Fig. S2B). Correspondingly, the enzymatic activities of PEPCK and G6Pase were also enhanced in an MOI-dependent manner (Fig. 2C and D; Fig. S2C and D). By contrast, no significant changes were observed in the mRNA expression of glycolysis-related genes, such as glucose transporter 2 (*GLUT2*), phosphofructokinase 1 liver isoform (*PFKL*), and triosephosphate isomerase 1 (*TPI1*), in SFTSV-infected cells compared to uninfected controls (Fig. 2B; Fig. S2E).

To further validate our findings, we examined the effect of SFTSV infection on glucose homeostasis in liver organoids derived from primary human hepatocytes (PHHs). The liver organoids effectively mimic the structural and physiological characteristics as well as basic tissue-level functions of native liver tissue (15). Our initial analysis demonstrated that liver organoids were susceptible to SFTSV infection, leading to the production of infectious virions (Fig. 2E and F). Moreover, SFTSV-infected liver organoids exhibited significant increases in glucose levels, *PCK2* mRNA expression, and PEPCK enzymatic activity when compared to uninfected controls (Fig. 2G through I).

Furthermore, we conducted an *in vivo* experiment using C57BL/6J mice pretreated with anti-interferon α receptor 1 (IFNAR1) immunoglobulin G (IgG) antibody and infected with SFTSV (16), which exhibited a significant elevation in blood glucose levels at both 1 and 3 days post-infection (dpi) (Fig. 2J). Consistent with our *in vitro* findings, hepatic PEPCK and G6Pase activities were significantly upregulated in SFTSV-infected mice (Fig. 2K and L), demonstrating a positive correlation with viral loads (Fig. 2M and N). Overall, these findings indicate that SFTSV exerts a pro-gluconeogenic effect by activating crucial enzymes involved.

To explore the impact of high glucose on SFTSV infection, we exposed SFTSV-infected Huh7 cells to varying concentrations of glucose. High glucose concentrations resulted in a significant and dose-dependent increase in intracellular levels of SFTSV RNA and viral titers in the supernatant (Fig. 2O and P). A similar enhancement was also observed in HepG2 cells; for instance, when the glucose concentration was increased from 5 mM to 40 mM, the viral titer in the supernatant rose by approximately 0.5 logs (Fig. S3A through C). Notably, regarding HUVECs, an endothelial cell line that exhibits weaker sensitivity to SFTSV compared to hepatocyte cells, the impact of high glucose on SFTSV infection is even more pronounced (Fig. S3D through F). Considering the pivotal role of AMP-activated protein kinase (AMPK) in glucose sensing, cell physiology, and innate immunity (17, 18), we hypothesized that AMPK might mediate the heightened susceptibility to SFTSV infection under conditions of high glucose. Immunoblotting analysis showed an MOI-dependent elevation in AMPK phosphorylation at Thr172 during SFTSV infection across different glucose conditions ranging from glucose free (GF), low glucose (LG), to high glucose (HG) (Fig. 2Q; Fig. S4). Furthermore, high glucose attenuated the AMPK phosphorylation induced by SFTSV infection (Fig. 2R). Notably, autophagy, which is crucial for optimal SFTSV propagation (19), was suppressed under conditions of high glucose as evidenced by a decreased ratio of lipidated LC3-II to non-lipidated LC3-I forms (Fig. 2R), suggesting that high levels of glucose facilitate SFTSV replication through alternative pathways regulated by AMPK, rather than autophagy. The antiviral effect exerted by AMPK through upregulation of IFN-β and IFN-stimulated genes (ISGs) was also compromised, as evidenced by decreased expression of *IFNB* and *ISG15* with increasing concentrations of glucose in SFTSV-infected Huh7 cells. (Fig. 2S and T). This suppression of IFN-I responses was also observed in HepG2 and HUVECs (Fig. S5). Taken together, these results indicate that high levels of glucose promote SFTSV infection by inhibiting AMPK activity and subsequent IFN-I responses.

### Underlying diabetes is associated with increased risk for the fatal outcome of SFTSV infection in females

**Fig 3:**
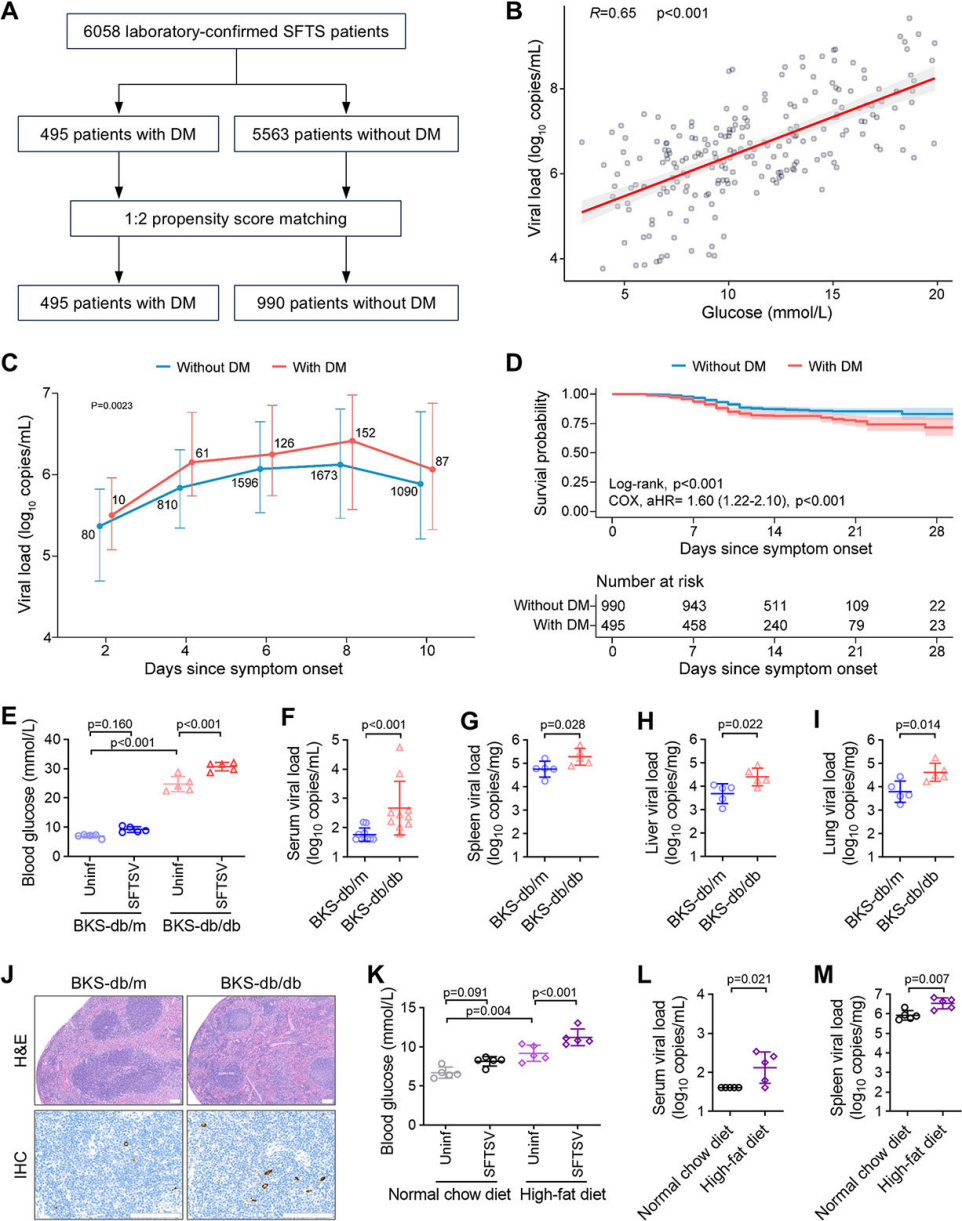
Underlying diabetes is associated with increased risk for enhanced viremia levels and increased fatality of SFTSV infection. (**A**) Flowchart of the recruitment and grouping process for SFTS patients. Among 6,058 laboratory-confirmed SFTS patients recruited, 495 patients with underlying diabetes and 990 patients without underlying diabetes matched by propensity score matching were included for analysis. (**B**) Pearson correlation coefficient between blood glucose levels and SFTSV viral loads in SFTS patients. (**C**) Dynamic profiles of SFTSV viral loads between patients with diabetes and those without diabetes. Data were presented as median and interquartile range. A GEE model was performed to consider the effect of age, sex, and onset-to-admission interval. The number of SFTS patients included for analysis at each time point was added to the line graphs. (**D**) Analysis of underlying diabetes on survival probability. The Kaplan-Meier method was used to analyze time-to-event data. Adjusted HR and 95% CI were conducted by a multivariable COX regression adjusted for age, sex, onset-to-admission interval, and pre-existing comorbidities. (**E**) Blood glucose levels of uninfected or SFTSV-infected BKS-db/bd and BKS-db/m mice at 3 days post-infection (dpi) (*n* = 5 per group). (**F–I**) Viral loads in serum (F; *n* = 10 per group) and tissue samples (*n* = 5 per group), including spleen (**G**), liver (**H**), and lung (**I**), collected from SFTSV-infected BKS-db/bd and BKS-db/m mice at 3 dpi. (**J**) Representative images from three biologically independent samples of spleen sections from SFTSV-infected BKS-db/bd and BKS-db/m mice at 3 dpi, stained with H&E or a rabbit polyclonal antibody against SFTSV NP. (**K**) Blood glucose levels of uninfected or SFTSV-infected normal chow diet and high-fat diet fed mice at 3 dpi (*n* = 5 per group). (**L, M**) Viral loads in serum (**L**) and spleen (**M**) from SFTSV-infected normal chow diet and high-fat diet fed mice at 3 dpi (*n* = 5 per group). Data were presented as mean ± s.d. The two-sided *P* values were examined using the Wilcoxon test (**F**) or Student’s *t* test (**G–I, L, M**) for comparison of variables between two groups, or using a two-way ANOVA multiple comparisons test for comparison of continuous variables among multiple groups (**E, K**).

Diabetes mellitus (DM) represents a metabolic disorder characterized by the inability of patients to maintain blood glucose levels below defined threshold values. We aimed to investigate whether individuals infected with SFTSV and having underlying DM experience more severe manifestations and poorer clinical outcomes. Among the cohort of 6,058 laboratory-confirmed SFTS patients, we designated 495 cases with underlying DM, while including 990 cases without DM as controls, matched through a 1:2 propensity score by controlling for age, sex, delay, and hypertension (Fig. 3A). We observed a significant positive correlation between blood glucose levels and serum viral loads among these SFTS patients (*R* = 0.65, *P* < 0.001; Fig. 3B), with the DM group consistently exhibiting higher viral loads throughout hospitalization compared to the non-DM group (*P* = 0.023, GEE model; Fig. 3C). Moreover, DM patients showed increased frequencies of dyspnea (9.3% vs. 6.0%, *P* = 0.024) and neurological symptoms, including confusion (22.6% vs. 15.9%, *P* = 0.002), coma (11.7% vs. 7.6%, *P* = 0.005), and convulsion (22.2% vs. 16.3%, *P* = 0.005) (Table S2). In addition, more severe biochemical abnormalities, including decreased albumin levels and increased lactate dehydrogenase and blood urea nitrogen concentrations, were present in DM patients (all *P* < 0.05; Table S3). Notably, the fatality rate among DM patients was significantly higher than that observed in non-DM patients (19.2%, 95/495 vs 12.4%, 123/990; *P* = 0.001). This association remained significant upon multivariable COX regression analysis, with an adjusted HR of 1.60 (95% CI, 1.22–2.10; *P* < 0.001; Fig. 3D). In the stratified analysis by sex, we observed females with underlying DM exhibited significantly enhanced viral loads (*P* < 0.001) and increased fatality rates (*P* < 0.001, log-rank test; adjusted HR = 1.95, 95% CI 1.40–2.71, *P* < 0.001, COX regression analysis) compared to their non-DM counterparts, whereas no such significant difference was noted among males with diabetes (Fig. S6). Taken together, these results demonstrate an elevated risk of death in female diabetic patients.

Next, we assessed the impact of underlying diabetes on SFTSV infection and disease pathogenesis in mouse models. We used female leptin receptor-deficient C57BLKS/J-*Lepr^db^/Lepr^db^* mice (BKS-db/db) as a genetic model of type 2 diabetes (T2D) (20), with female non-diabetic mice (BKS-db/m) serving as controls. Both mouse models exhibited elevated blood glucose levels following SFTSV infection at 3 dpi, with only the BKS-db/db model showing statistical significance (Fig. 3E). Notably, BKS-db/db mice exhibited significantly higher viral loads in serum and tissues (spleen, liver, and lung) compared to BKS-db/m mice (Fig. 3F through I). More pronounced pathological alterations were observed in the spleens of SFTSV-infected BKS-db/db mice, such as white pulp atrophy as revealed by hematoxylin and eosin (H&E) staining (Fig. 3J).

To corroborate these findings, we also employed a non-genetic T2D mouse model created by a combination of a high-fat diet and a low-dose streptozotocin treatment (20). As anticipated, mice fed with a high-fat diet exhibited elevated levels of blood glucose that further increased upon SFTSV infection (Fig. 3K). Consistent with the genetic model, these mice also showed higher viral loads in serum and spleen samples compared to mice fed with a normal chow diet (Fig. 3L and M), supporting the role of diabetes in exacerbating SFTSV infection and disease pathogenesis.

### Metformin provides beneficial effects in treating SFTS patients with underlying diabetes or hyperglycemic complications

We sought to evaluate the impact of two commonly used glucose-lowering drugs, metformin and insulin, on the clinical outcomes of SFTS patients. Among 495 SFTS patients with pre-existing DM, 90 received combined metformin and insulin treatment, 327 received insulin treatment alone, 14 received metformin treatment alone, and 64 patients did not receive either medication. Through performing multivariable COX regression analysis, we found that patients receiving combined metformin and insulin treatment had a significantly higher survival rate (91.1%) compared to those treated with insulin alone (78.6%) or without any glucose-lowering drugs (73.4%). Interestingly, there was no significant difference in survival rates between the patients treated with insulin alone and those without any glucose-lowering drugs (Fig. S7A). This finding suggests that metformin exerts a beneficial effect on SFTS patients with DM independent of insulin therapy.

**Fig 4:**
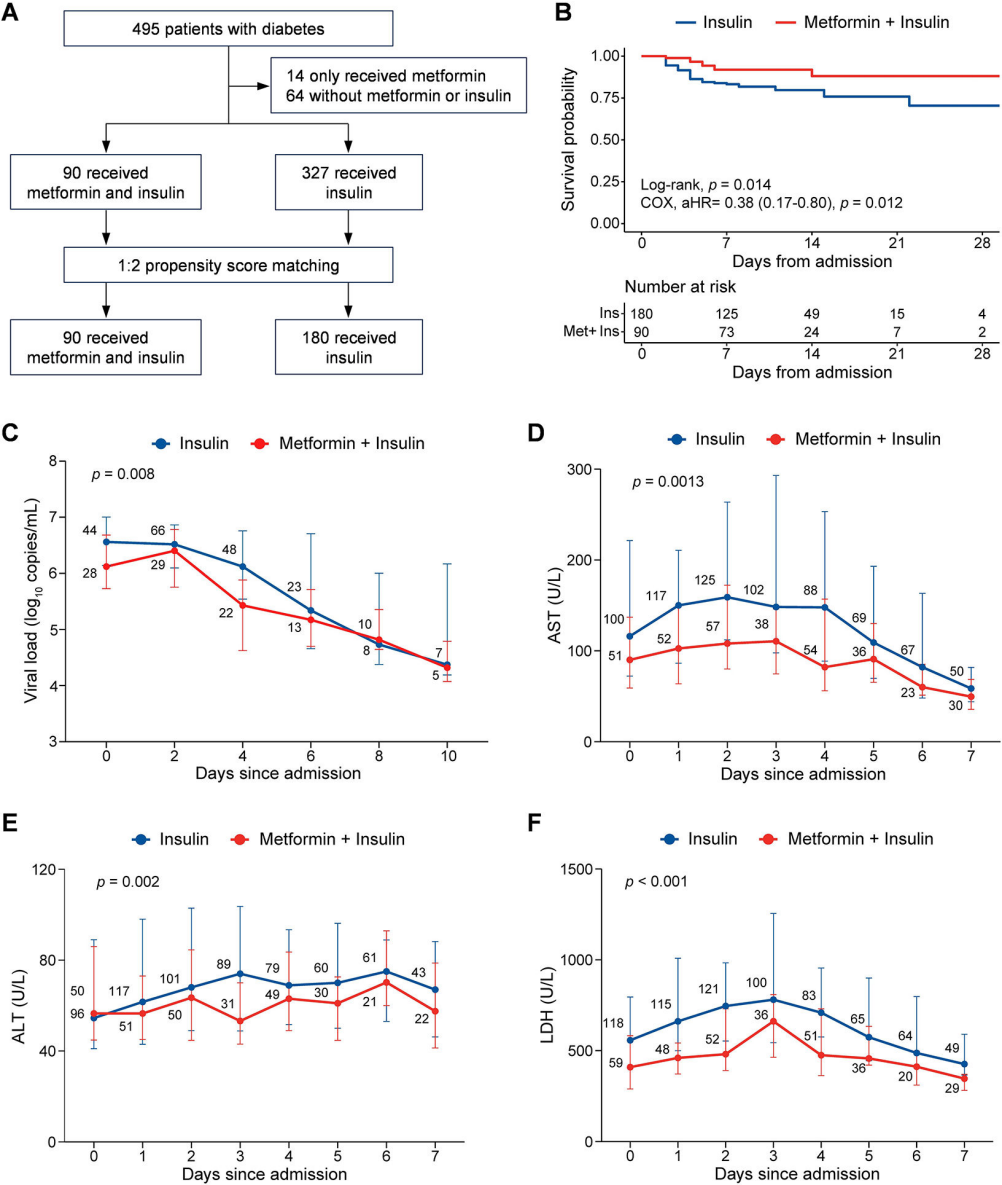
Clinical efficacy of metformin in treating SFTS patients with underlying diabetes. (**A**) Flowchart of the recruitment and grouping process for SFTS patients. Among 495 laboratory-confirmed SFTS patients with underlying diabetes recruited, 90 patients receiving combined metformin and insulin and 180 patients receiving insulin treatment alone by propensity score matching were included for analysis. (**B**) Analysis of metformin treatment on the survival probability of SFTS patients. The Kaplan-Meier method was used to analyze time-to-event data. Adjusted HR and 95% CI were conducted by a multivariable COX regression adjusted for age, sex, onset-to-admission interval, and pre-existing comorbidity. (**C–F**) Dynamic profiles of SFTSV viral load (**C**) and levels of AST (**D**), ALT (**E**), and LDH (**F**), between patients receiving combined metformin and insulin and those receiving insulin treatment alone. Data were presented as median and interquartile range. A GEE model was performed to consider the effect of age, sex, and onset-to-admission interval. The number of SFTS patients included for analysis at each time point was added to the line graphs.

To further validate the therapeutic efficacy of metformin, we conducted a propensity score matching analysis, by pairing 90 patients receiving combined metformin and insulin with 180 patients receiving only insulin at a 1:2 ratio (Fig. 4A). Both groups were well-balanced in terms of demographic characteristics, clinical manifestations, and important laboratory indicators upon admission (Table S4). After adjusting for confounding factors, combination therapy using metformin and insulin significantly increased the survival rate (91.1%) compared to monotherapy using only insulin (79.4%), yielding an adjusted HR of 0.38 (95% CI, 0.17–0.80; *P* = 0.014; Fig. 4B). Furthermore, GEE modeling analysis demonstrated more rapid clearance of viremia (*P* = 0.008) and faster recovery from abnormal laboratory parameters, including aspartate aminotransferase (AST; *P* = 0.001), alanine aminotransferase (ALT; *P* = 0.002), and lactate dehydrogenase (LDH; *P* < 0.001), among patients receiving the combination therapy (Fig. 4C through F). However, the combination therapy did not exhibit superior effects on glucose-level dynamics compared to insulin monotherapy (Fig. S7B), suggesting that metformin’s therapeutic benefit against SFTS may involve mechanisms other than solely glucose lowering. Furthermore, we found that the metformin’s therapeutic efficacy was not influenced by sex, exhibiting an adjusted HR of 0.13 (95% CI, 0.02–0.98; *P* = 0.047) for males and 0.39 (95% CI, 0.17–0.89; *P* = 0.026) for females (Fig. S8A and B). Consistently, the viral loads and liver function indicators were significantly reduced in the combination therapy group compared to the insulin monotherapy group, irrespective of sex (Fig. S8C through J). In addition, our analysis revealed no deaths among 19 SFTS patients with hyperglycemia but without DM who received metformin treatment, while 23.7% (14/59) of matched non-metformin-treated patients succumbed to the disease (Fig. S9). These findings further emphasize the potential therapeutic role of metformin in SFTS, potentially extending beyond its glucose-lowering effect.

### Metformin inhibits the replication of SFTSV through suppressing autophagy via modulation of the AMPK-mTOR pathway

**Fig 5:**
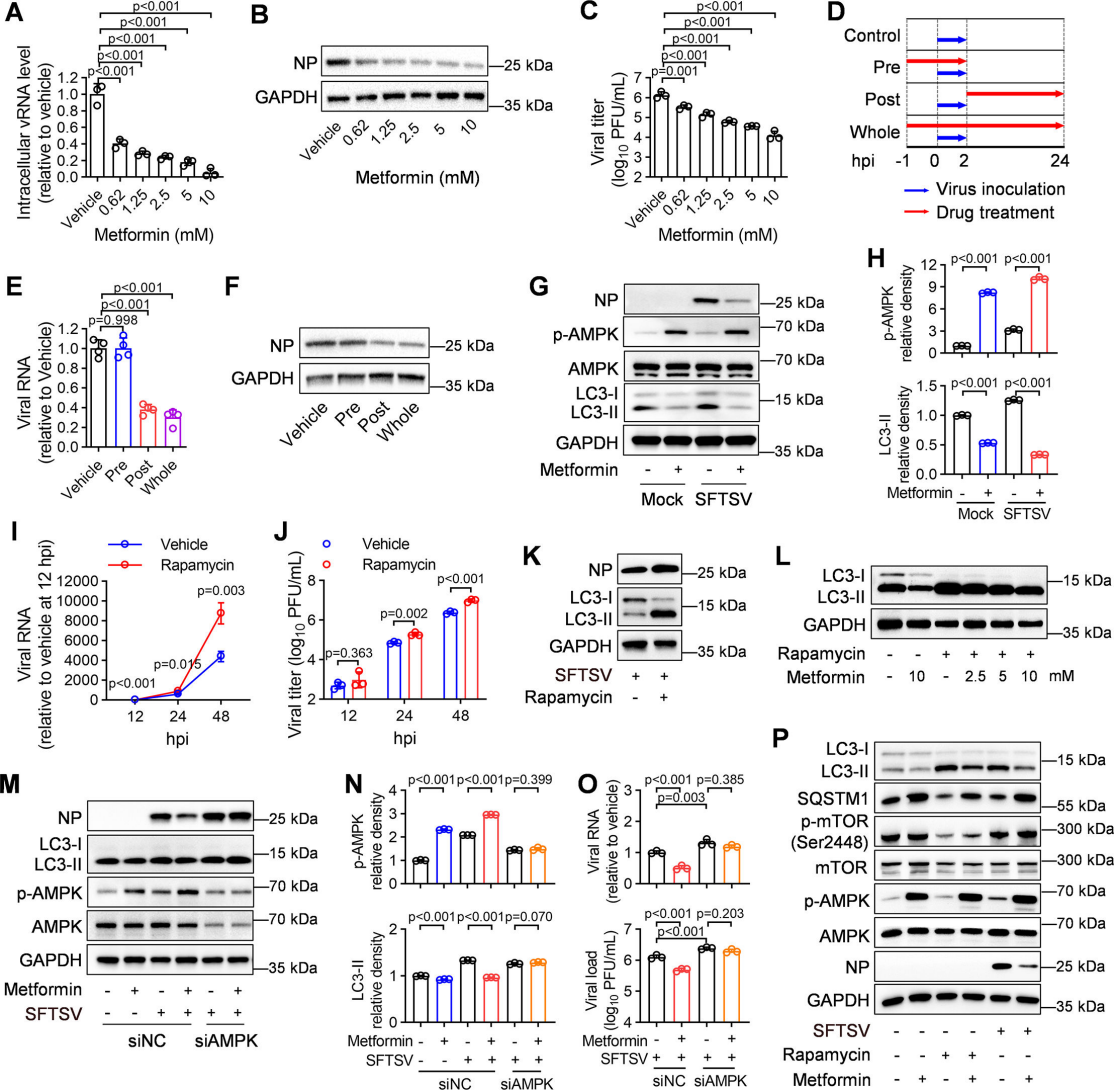
Metformin suppresses autophagy via modulation of the AMPK-mTOR pathway to inhibit SFTSV replication. (**A–C**) Intracellular SFTSV RNA level (**A**), NP levels (**B**), and supernatant viral titers (**C**) were measured in SFTSV-infected Huh7 cells (MOI = 1) cultured with indicated concentrations of metformin at 24 hpi; *n* = 3 biologically independent samples. (**D**) Schematic diagram of the time-of-addition assay of metformin. (**E, F**) Huh7 cells infected with SFTSV (MOI = 0.1) for 2 h were treated with vehicle (control) or metformin in pre-infection, post-infection, or whole time. Intracellular SFTSV RNA levels (**E**) and NP levels (**F**) were measured at 24 hpi; *n* = 4 biologically independent samples. (**G, H**) SFTSV-infected Huh7 cells (MOI = 1) cultured with DMEM containing 20 mM glucose were treated with metformin (5 mM) for 24 h. Whole-cell extracts (WCEs) were analyzed by immunoblotting analysis using the indicated antibodies (**G**). The relative density of p-AMPK and LC3-II was compared among groups (**H**). (**I, J**) Intracellular SFTSV RNA level (**I**) and supernatant viral titers (**J**) were measured in SFTSV-infected Huh7 cells (MOI = 0.1) treated with rapamycin (10 µM) at 12, 24, and 48 hpi; *n* = 3 biologically independent samples. (**K**) WCEs from rapamycin-treated Huh7 cells at 24 hpi were analyzed by immunoblotting analysis using the indicated antibodies. (**L**) WCEs from Huh7 cells treated with rapamycin (10 µM) and metformin for 24 h were analyzed by immunoblotting analysis using the indicated antibodies. (**M–O**) AMPK-knockdown Huh7 cells were infected with SFTSV (MOI = 1) and treated with metformin. Indicated proteins in WCEs (**M**), intracellular SFTSV RNA levels, and supernatant viral titers (**O**) were measured at 24 hpi; *n* = 3 biologically independent samples. The relative density of p-AMPK and LC3-II was compared among groups (**N**). (**P**) WCEs from SFTSV-infected or rapamycin-treated Huh7 cells in the presence or absence of metformin were analyzed by immunoblotting analysis using the indicated antibodies. Data were presented as mean ± s.d. The two-sided *P* values were examined using Student’s *t* test for comparison of variables between two groups (**I, J**), one-way ANOVA followed by Tukey’s multiple comparisons test (**A, C, E, N**), or two-way ANOVA for comparison of continuous variables among multiple groups (**H, O**). The presented images are representative of three independent experiments in immunoblotting analysis (**G, K, L, M, P**).

We initially investigated the potential of metformin to inhibit SFTSV infection *in vitro*. Huh7 cells were infected with SFTSV and subsequently treated with varying concentrations of metformin. At 24 hours post-infection (hpi), metformin significantly suppressed SFTSV infection, as shown by reduced levels of intracellular SFTSV RNA and nucleocapsid protein (NP), along with decreased viral titers in the supernatant, which were determined using RT-qPCR, immunoblotting analyses, and immunological focus assay, respectively (Fig. 5A through C). Furthermore, a dose-dependent inhibitory effect on SFTSV infection was observed for metformin treatment in Huh7 cells, HepG2 cells, and HUVECs (Fig. 5A through C; Fig. S10A through F). Metformin, at concentrations below 10 mM, exhibited no notable cytotoxic effects on these cell lines as determined by CCK-8 analysis (Fig. S10G through I).

We subsequently investigated the mechanism underlying metformin’s inhibitory effect on SFTSV infection. Initially, we assessed whether metformin had a virucidal effect on intact virions of SFTSV by incubating them at specified concentrations for 1 hour or subjecting them to heat treatment prior to inoculation into Huh7 cells. Our analysis showed that intracellular levels of SFTSV RNA were comparable between the mock-treated and metformin-treated groups, while the heat-treated SFTSV group exhibited background levels of viral RNA at 24 hpi (Fig. S10J), indicating that metformin treatment does not exert a virucidal effect on SFTSV. Subsequently, we conducted a time-of-addition assay to determine which stage of SFTSV infection is targeted by metformin (Fig. 5D). Metformin was added either before or after SFTSV infection, and infected cells were harvested at 24 hpi. By quantifying intracellular viral RNA and NP levels, we observed that metformin inhibited both the post-entry phase (Post) and the entire infection process (Whole), but had no such effect during the entry process (Fig. 5E and F). Collectively, these results suggest that metformin exerts its anti-SFTSV efficacy after viral entry.

Next, we aimed to elucidate the mechanism underlying the inhibitory effect of metformin on SFTSV infection. Huh7 cells were infected with SFTSV or mock-infected and treated with metformin in high glucose (HG, 20 mM) media. Immunoblotting analysis revealed that metformin enhanced AMPK phosphorylation under HG conditions (Fig. 5G and H). However, unlike glucose’s regulation of IFN-I response through AMPK, metformin, as an AMPK agonist, exhibited minimal impact on the expression of *IFNB* and *ISG15* in SFTSV-infected Huh7 cells, HepG2 cells, and HUVECs (Fig. S11A through F), instead reducing the expression of LC3-II, a marker of autophagy, under HG conditions (Fig. 5G and H). To further explore the relationship between autophagy and SFTSV replication, we treated Huh7 cells with rapamycin, a known inducer of autophagy, which showed a significant increase in intracellular SFTSV RNA levels, supernatant viral titers, and intracellular NP levels (Fig. 5I through K), indicating that rapamycin-induced autophagy flux promotes virus replication. Furthermore, we observed a dose-dependent reduction in the LC3-II/I ratio in rapamycin-treated Huh7 cells upon treatment with metformin (Fig. 5L). Collectively, these results suggest that metformin may influence SFTSV infection by modulating the AMPK and autophagy pathways.

For further validation, we silenced AMPK expression in Huh7 cells using siRNA at a concentration of 80 nM, which did not show significant cell cytotoxicity (Fig. S12). AMPK knockdown resulted in a significant reduction of metformin’s ability to suppress autophagy and SFTSV infection (Fig. 5M through O). Meanwhile, the neutral effect of metformin on IFN-I responses remained unchanged following AMPK knockdown (Fig. S13). We also observed similar effects of AMPK knockdown on metformin’s anti-SFTSV activity in HUVECs (Fig. S14A through D). Moreover, treating Huh7 cells with the AMPK agonist, AICAR, resulted in a significant suppression of viral replication (Fig. S15A through D). Similar to metformin, AICAR decreased the expression of LC3-II as well (Fig. S15E and F). Considering the significance of the AMPK-mTOR pathway in regulating autophagy, we measured the effects of metformin on phosphorylated mTOR, a crucial autophagy pathway regulator (21), and SQSTM1, an autophagosome adapter and marker of autophagic flux (22), during SFTSV infection or rapamycin treatment. Our analysis showed that metformin increased levels of SQSTM1 and phosphorylated mTOR, while reducing the LC3-II/I ratio in both SFTSV-infected and rapamycin-treated Huh7 cells (Fig. 5P). Similar changes in autophagic markers were also observed in metformin-treated HUVECs (Fig. S14E). Furthermore, insulin showed no significant effect on SFTSV infection in Huh7 and HepG2 cells (Fig. S16). These findings indicate that metformin potentially inhibits SFTSV infection by suppressing autophagy via modulation of the AMPK-mTOR pathway.

### Metformin treatment protects BKS-db/db mice from lethal SFTSV infection

**Fig 6:**
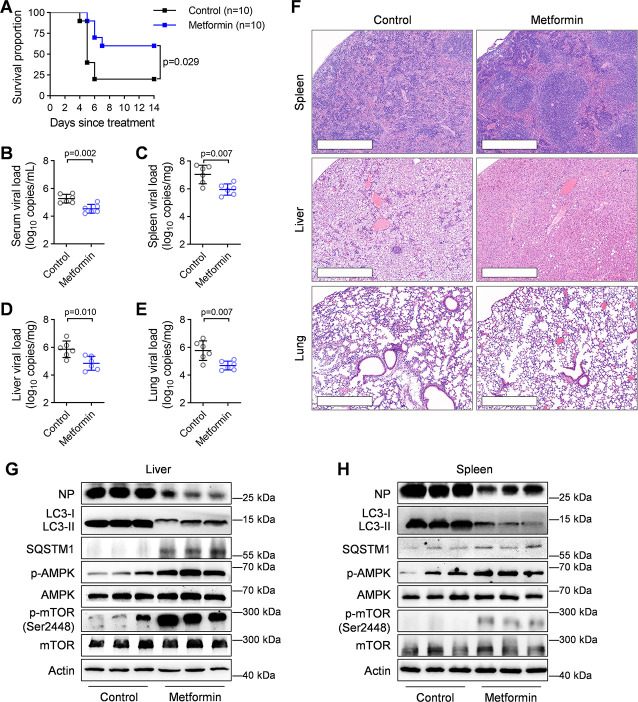
Treatment effectiveness of metformin against lethal SFTSV infection in BKS-db/db mice. (**A**) Survival curves of metformin- or vehicle-treated BKS-db/bd mice (*n* = 10 per group) that were pretreated with anti-IFNAR1 IgG antibody and infected with SFTSV (4 × 10^4^ PFU per mouse). The Kaplan-Meier method was used to analyze time-to-event data. (**B–E**) Viral loads in serum (**B**), spleen (**C**), liver (**D**), and lung (**E**) from SFTSV-infected SK-db/bd mice with or without (control group) metformin treatment at 5 days post-infection (dpi) (*n* = 6 per group). Data were presented as mean ± s.d. The two-sided *P* values were examined using Student’s *t* test. (**F**) Representative hematoxylin and eosin (H&E) images from three biologically independent samples of spleen, liver, and lung sections from SFTSV-challenged BKS-db/bd mice with or without metformin treatment at 5 dpi. Scare bar: 500 µm. (**G, H**) Liver (**G**) and spleen samples (**H**) from SFTSV-challenged BKS-db/bd mice with or without metformin treatment at 5 dpi were analyzed by immunoblotting with the indicated antibodies (*n* = 3 biologically independent samples).

The therapeutic efficacy of metformin against SFTSV infection was further assessed in BKS-db/db mice with a lethal phenotype induced by pretreatment with anti-IFNAR1 antibody. To simulate prolonged use of metformin in diabetes management, the mice were administered metformin via a stomach probe for 10 days, including 5 days prior to SFTSV infection and 5 days during viral infection. Compared to the vehicle-treated group, metformin treatment significantly reduced fatality rate (80%, 8/10 versus 40%, 4/10; *P* = 0.029, log-rank test; Fig. 6A) and decreased viral loads in serum and tissue samples (spleen, lung, and liver) (Fig. 6B through E). Furthermore, metformin treatment alleviated histopathological changes, including the loss of white pulp accompanied by increased megakaryocyte counts in the spleen, mononuclear cell infiltration, and hepatocyte ballooning degeneration in the liver, as well as interstitial infiltrates and extensive alveolar thickening in the lung (Fig. 6F). Consistent with our previous *in vitro* findings, immunoblotting analysis of liver and spleen tissues revealed that metformin treatment enhanced AMPK and mTOR phosphorylation while increasing SQSTM1 expression and concurrently inhibiting autophagy and SFTSV infection in BKS-db/db mice (Fig. 6G and H). Collectively, these results support considering metformin as a promising therapeutic candidate for SFTSV infection, particularly in diabetic individuals.

## DISCUSSION

A long-standing belief suggests that viral infections can disrupt the glucose metabolism of the hosts (23). The main argument is that persistent infections caused by viruses such as coxsackievirus B and hepatitis C virus have been associated with the development of diabetes (8, 24). Notably, even acute viral infections can initially cause dysglycemia, including hyperglycemia and hypoglycemia, potentially leading to serious complications. For instance, a retrospective analysis of 453 patients with confirmed SARS-CoV-2 infection revealed that 28.5% experienced hyperglycemia, which was correlated with a higher all-cause mortality rate compared to normoglycemic controls (25). Conversely, during acute Ebola virus infection, a highly pathogenic agent causing severe and systemic disease, both severe hypoglycemia and hyperglycemia have been observed, with severe hypoglycemia serving as a risk factor for mortality (26). Overall, abnormalities in glucose metabolism induced by acute viral infections may vary across different viruses, necessitating further clinical data to corroborate this observation. Our current multicenter longitudinal study has successfully recruited a substantial cohort of 6,764 patients with laboratory-confirmed SFTSV infection from five sentinel hospitals in China spanning the period from 2010 to 2023. Through our comprehensive analysis, we discovered that SFTSV infection triggers hyperglycemia in patients without pre-existing diabetes, and importantly, demonstrated that hyperglycemia serves as an independent risk factor for elevated viremia levels and increased fatality rates among SFTS patients.

Considering the susceptibility of human hepatocytes to SFTSV (14), we hypothesized that the virus potentially influences glucose metabolism by modulating hepatic glucose production, which involves glycogen breakdown (glycogenolysis) and *de novo* synthesis of glucose from noncarbohydrate precursors (gluconeogenesis) (2). Through conducting *in vitro* infections of Huh7 and HepG2 cells, as well as *ex vivo* infections of PHHs-derived liver organoids, we demonstrated that SFTSV infection promotes gluconeogenesis by upregulating the activity of key enzymes such as PEPCK and G6P, without significant alterations in glycolysis. Similar observations have been reported in hepatocytes infected with SARS-CoV-2, where increased glucose production was associated with induction of PEPCK activity (5), potentially due to enhanced production and secretion of Golgi protein 73 (GP73) (27). SARS-CoV-2 infection has also been shown to induce insulin resistance and impair β-cell function, leading to potential exhaustion of β-cells and hyperglycemia (28). However, our current study has not explored the impact of SFTSV infection on the hormone insulin, necessitating further studies to elucidate the detailed mechanisms underlying SFTSV-induced hepatic gluconeogenesis. Furthermore, although our *in vitro* experiments showed that SFTSV does not affect glucose transport or glycolysis, SFTSV-associated hyperglycemia may also be attributed to a deficiency in the insulin-GLUT4 pathway. This hypothesis is supported by evidence: first, SFTSV antigen has been detected in heart and pancreatic tissues of SFTS patients (29); second, several RNA viruses have been shown to induce GLUT4-mediated glucose influx and disposal (30). In addition, our *in vitro* experiments revealed certain inconsistencies among the two liver cell lines and endothelial cells, which may be related to their differing susceptibilities to SFTSV (14), and various responses under high glucose or metformin treatment conditions (31, 32). Notably, we selected actin as the loading control protein for immunoblotting analysis conducted under different glucose concentrations, as such conditions may affect GAPDH expression (33).

Diabetes, a chronic metabolic syndrome, is characterized by impaired glucose utilization and elevated glycemic levels. This condition has been associated with a heightened susceptibility to infectious diseases and an increased risk of infection-related complications. For instance, pre-existing diabetes exacerbates the progression of respiratory viral infections, increasing the risk of severe outcomes (28, 34, 35). Notably, diabetes confers a greater risk for complications and mortality in females compared with males (36). A meta-analysis involving 5.16 million participants revealed a 13% higher all-cause mortality rate among female diabetes patients than their male counterparts (37). Our current study demonstrates that underlying diabetes is associated with an increased risk of fatal outcomes from SFTSV infection in females, contrasting with males. This seems to be related to the significant impact of sex hormones on immunological and inflammatory responses (38), which are crucial in determining the prognosis of SFTSV infection (39). Furthermore, estrogens have been implicated in glucose tolerance and insulin sensitivity (40). However, the detailed mechanisms underlying the adverse outcomes observed in diabetic females following viral infections warrant further exploration. Furthermore, since no notable disparities in SFTSV infection between male and female mice were previously observed, sex was not considered in our animal experiments. Instead, females were empirically used.

Insulin therapy and metformin are commonly prescribed for diabetes management; however, their impact on clinical outcomes in patients with acute viral infections has rarely been investigated. For COVID-19 patients with diabetes, insulin therapy has been associated with increased mortality (41, 42), whereas metformin initially exhibited a therapeutic potential during the early stage of the pandemic (43, 44), possibly due to its antiviral and anti-inflammatory properties (44, 45). However, subsequent randomized clinical trials failed to confirm metformin’s efficacy in viral clearance, prevention of severe complications, or reduction in mortality rates (46, 47). Our current study, based on a large-scale multi-center observation, revealed a substantial correlation between pre-existing diabetes and elevated viremia, as well as increased fatality rates in SFTS, after accounting for confounding factors. In addition, in both genetic and non-genetic mouse models of diabetes, we found that pre-existing diabetes promoted SFTSV replication and pathogenesis. The persistent high blood glucose levels associated with diabetes can intricately modulate immune responses, increasing susceptibility to viral infections (48). For instance, hyperglycemia impairs costimulatory molecule expression, antigen transport, and T-cell priming in specific subsets of lung dendritic cells, resulting in a defective antiviral adaptive immune response, delayed viral clearance, and increased mortality (49). In addition to affecting adaptive immunity, our current study has revealed that elevated glucose levels suppressed AMPK activation, which subsequently inhibits the IFN-I response and promotes accelerated replication of SFTSV in virus cell lines. AMPK activation has been shown to directly phosphorylate TBK1, triggering the recruitment of IRF3 and assembly of MAVS or STING signalosomes, thereby enhancing innate antiviral immunity (18). Moreover, glucose restriction may reduce lactate production and relax lactate-mediated inhibition of MAVS, IRF3, and NF-κB signaling, enhancing the IFN-I response and impairing viral infection (50, 51). However, our study observed no significant changes in mRNA expression levels of glycolysis-related genes, nor measured lactate levels upon SFTSV infection. Therefore, further investigation is needed to understand the role of lactate in the mechanism by which hyperglycemia promotes SFTSV infection. Furthermore, we observed that high glucose suppresses autophagy, a process involved in the propagation of SFTSV (19). It is hypothesized that, under high glucose conditions, the impact of IFN-I responses on viral replication at the cellular level may be more pronounced than that of autophagy. In addition, there may be a crosstalk between IFN-I signaling and autophagy that contributes to virus elimination (52).

To further assess the therapeutic effect of metformin, we performed a large cohort study on SFTS patients, demonstrating that metformin significantly reduced viremia and decreased SFTSV-related deaths among individuals with pre-existing diabetes, in contrast with the disadvantageous effect of insulin administration. Our *in vitro* examination further revealed that metformin suppressed SFTSV replication in hepatocytes and endothelial cells. Previous studies have shown the ability of metformin to inhibit infections caused by the Zika virus in microglia and the hepatitis C virus in OR6 cells by activating IFN-I signaling (53, 54). However, our study found no significant increase in the expression of *IFN*B and *ISG15*, suggesting that the anti-SFTSV effect of metformin may not be mediated through this pathway. Similarly, metformin has been shown to inhibit influenza A virus replication independent of IFN-α production in THP-1 cells (55). Furthermore, our observations indicated that metformin treatment activated AMPK in cells. The variability in AMPK-regulated IFN-I responses may be related to the different types of viruses and cells involved (56, 57).

Accumulating evidence has recently indicated a crucial role for autophagy in mediating the pharmacological actions of metformin (58, 59). According to a previous study, SFTSV could induce autophagy flux and exploit this cellular degradation process for its life cycle (19). Our study consistently demonstrated that pretreatment with rapamycin, an autophagy agonist, facilitated SFTSV infection in cells. However, despite metformin’s ability to induce autophagy through AMPK activation (59), our analysis showed that metformin, acting as AMPK agonist, prevented SFTSV-induced autophagic flux. This was evidenced by reduced LC3-II, increased SQSTM1, and phosphorylated mTOR levels. Suppression of AMPK through RNAi weakened metformin’s inhibitory effects on autophagy and antiviral activities. Furthermore, AICAR, another AMPK agonist, also inhibited autophagy induced by SFTSV infection or rapamycin treatment. Similar observations have been reported in neurons and neuroblastoma cells, where both metformin and AICAR activated AMPK, resulting in a slight decrease in autophagic flux (60). In addition, metformin-mediated inhibition of autophagy has been shown in endothelial cells cultured under high glucose conditions through activation of the Hedgehog signaling pathway (61). Beyond this *in vitro* effect, our animal experiments revealed that metformin inhibited viral multiplication by suppressing autophagic flux, which mitigated pathological damage and increased survival rates of diabetic mice infected with SFTSV. Notably, all 19 hyperglycemic patients receiving metformin treatment survived SFTSV infection, while 23.7% of the matched patients who did not receive metformin succumbed to the disease. These findings collectively validated the clinical efficacy of metformin through a plausible biological pathway.

In summary, our multicenter clinical study uncovered a higher risk of mortality among hyperglycemic or diabetic patients infected with SFTSV. We also demonstrated the antiviral effectiveness of metformin in these high-risk populations. Further analysis using hepatic cell lines and liver organoids revealed that SFTSV infection triggers gluconeogenesis to facilitate viral replication. Both *in vitro* and animal studies demonstrated that metformin suppresses SFTSV replication, which is related to its inhibition of autophagy through the AMPK-mTOR pathway (Fig. S17). These findings highlight the promising therapeutic potential of metformin for treating SFTS, particularly among individuals with hyperglycemia and diabetes.

## MATERIALS AND METHODS

### Study design

The study aimed to investigate the impact of hyperglycemia and diabetes on the SFTSV infection outcome. To achieve this goal, we conducted a multicenter clinical study to examine the influence of hyperglycemia and diabetes on outcomes of SFTSV infection and evaluated the efficacy of glucose-lowering drugs in treating SFTSV infection. Furthermore, we utilized human cell lines and liver organoids to investigate the effect of SFTSV infection on glucose metabolism. We further assessed the impact of high glucose levels on SFTSV infection both *in vitro* and *in vivo*. In addition, we explored the mechanism underlying metformin’s antiviral effect and demonstrated its effectiveness in treating SFTSV infection using animal models.

Mice were randomly assigned to different groups, and no exclusion occurred during analyses. Data collection and analysis were not blinded. All experiments were replicated as indicated in the figure legends. Representative images for hematoxylin and eosin staining, immunohistochemistry, microscopy, and western blotting are from at least *n* = 3 independent sample preparations.

### Patients and sample collection

We conducted a multicenter retrospective clinical investigation in five large sentinel hospitals in China for treating SFTS (154th hospital in Xinyang, Henan Province; Yantai Qishan Hospital in Yantai, Shandong Province; Shandong Public Health Clinical Center in Jinan, Shandong Province; The First Affiliated Hospital of Nanjing Medical University in Nanjing, Jiangsu Province; and Nanjing Drum Tower Hospital in Nanjing, Jiangsu Province) from 2011 to 2023. Patients were confirmed with SFTSV infection if they tested positive for the detection of SFTSV using RT-qPCR. Patients with serious underlying conditions or hospitalized for less than 2 days were excluded from the analysis. Demographic data, medical history, clinical symptoms and signs, laboratory tests, treatment regimens, and clinical outcomes were extracted from electronic medical records by trained physicians using a standardized protocol and recorded in an EpiData database. Follow-up visits were conducted for patients who discontinued therapy due to clinical deterioration and left the hospital, to determine their final outcomes and date of death. The primary outcome assessed was SFTS-related fatality. Secondary outcomes included changes in viral loads, along with important laboratory parameters identified as risk factors for death.

### Definitions and analysis process

Hyperglycemia was defined as a blood glucose level of ≥7 mmol/L upon admission, as previously reported (13). We first analyzed the relationship between blood glucose levels and viral loads to assess the effects of SFTSV-induced hyperglycemia in patients without pre-existing diabetes. Then we evaluated the impact of hyperglycemia on mortality risk. In addition, we examined the association between pre-existing diabetes and both viral loads and mortality. Furthermore, we investigated whether metformin treatment influenced the prognosis and outcome of SFTS by comparing fatality rates and viremia levels in patients with pre-existing diabetes versus those without pre-existing diabetes but experiencing hyperglycemia.

### Cells and viruses

Huh7 and HepG2 cells obtained from China Center for Type Culture Collection (CCTCC), as well as human umbilical vein endothelial cells (HUVECs) and Vero cells obtained from American Type Culture Collection (ATCC), were cultured in Dulbecco’s modified Eagle’s medium (DMEM; Gibco, Cat. 11995065) supplemented with 10% fetal bovine serum (FBS; Gibco, Cat. 10099141) and 1% penicillin-streptomycin (Gibco, Cat. 15140122). All the cells were cultured at 37°C in a humidified atmosphere of 5% CO_2_.

SFTSV strain HBMC16_human_2015 was obtained from the National Virus Resource Centre. The virus was propagated in Vero cells and used appropriately in this study. Experiments with viruses were performed in a biosafety level 2 (BSL-2) or BSL-3 facilities, in accordance with the institutional biosafety operating procedures.

### Culture of primary human hepatocytes-derived organoids

PHHs-derived liver organoid was obtained from commercial sources (Beijing Daxiang Biotech, Cat. SP109620). After thawing, the cells were washed with 5 mL of coating solution (Beijing Daxiang Biotech, Cat. KC100143) and centrifuged at 400 × *g* for 5 min at 4°C. After two washes, the cell pellets were resuspended in liver organoid expansion medium (Beijing Daxiang Biotech, Cat. HG10010), mixed with Matrigel (Beijing Daxiang Biotech, Cat. MG100101) in a volume twice that of the expansion medium. The cell suspension was then seeded into a 24-well plate with 50 µL per well and incubated at 37°C until Matrigel solidified. Next, expansion medium containing 1× anti-apoptotic factor (Beijing Daxiang Biotech, Cat. IA100101) was added to each well, with a volume of 500 µL. The medium was replaced every 3–4 days to maintain optimal growth conditions. For organoids passaging, the liver organoids were dissociated into small fragments using organoid dissociation solution (Beijing Daxiang Biotech, Cat. KC100142) and centrifuged at 400 × *g* for 5 min at 4°C. The passaging process occurred once every 4–6 days using a split ratio ranging from 1:2 to 1:3. Small fragments were resuspended in a medium consisting of a 1:2 ratio of expansion medium to Matrigel and cultured in expansion medium containing 1 × anti-apoptotic factor. To induce differentiation, the organoids were cultured in expansion medium for 5 days before transferring to liver organoid differentiation medium. The fresh differentiation medium was replaced every 3 days.

### Glucose level measurement

Cell culture supernatant and serum samples from mice were collected for measurement of glucose levels using the Glucose Assay Kit with O-toluidine (Beyotime, Cat. S0201S). The glucose concentration in each sample was calculated according to the standard curve.

### Measurement of enzyme activities *in vitro*

SFTSV-infected Huh7 and HepG2 cells were collected and counted at 48 hpi. The enzyme activities of phosphoenolpyruvate carboxykinase (PEPCK) and glucose-6-phosphatase (G6Pase) were measured using commercial kits (Jiancheng Bioengineering Institute, Cat. A131-1-1; Comin Biotech Co., Ltd., Cat. G6P-1-Y). The liver tissues and liver organoids were homogenized in cold lysis buffer and centrifuged at 8,000 × *g* for 15 min, and the protein concentration of the supernatant was determined with a BCA Protein Quantification Kit (Vazyme, Cat. E112-01). Enzyme activities were analyzed as described above.

### Indirect immunofluorescence assay

SFTSV-infected liver organoids were collected at 48 hpi and fixed in 4% paraformaldehyde for 20 min, permeabilized in 0.5% Triton X-100 (Solarbio, Cat. T8200) in PBS for 30 min, and then blocked in 5% bovine serum albumin (BSA; Sigma, Cat. V900933) in PBST (0.1% Tween-20) for 30 min at room temperature. Then, liver organoids were stained with a rabbit polyclonal antibody against SFTSV NP (1:1,000) overnight at 4°C. Goat anti-rabbit IgG/APC antibody (Solarbio, Cat. K0034G-APC) was used at a dilution of 1:1,000 for 1 h. After washing three times with PBS, the nuclei were stained with DAPI, and the cell membrane was stained with Actin-Tracker Green-488 (Beyotime, Cat. C2201S). The fluorescence images were captured using a confocal laser-scanning microscope (DeltaVision Ultra, GE Healthcare).

### RNA isolation and RT-qPCR

Total RNA was extracted from cells, tissues and cell culture supernatant with the RNAprep pure Cell Kit (TIANGEN, Cat. DP430), RNA Easy Fast Tissue/Cell Kit (TIANGEN, Cat. DP451), and TIANamp Virus RNA Kit (TIANGEN, Cat. DP315-R), respectively. Intracellular levels of SFTSV RNA and mRNA of target genes were determined using RT-qPCR with the HiScript II One Step qRT-PCR SYBR Green Kit (Vazyme, Cat. Q221) in a LightCycler 480 (Roche). All data were normalized to the housekeeping gene Actin. Quantification of viral loads in cell culture supernatant or mouse tissue suspension was performed using RT-qPCR with the HiScript III U + One Step qRT-PCR Probe Kit (Vazyme, Cat. Q225) in a LightCycler 480. Sequences of primers and probes used in the study are shown in Table S5.

### Immunological focus assay

Cell culture supernatant or homogenized mouse tissue suspension was serially diluted 10-fold to incubate with Vero cells confluent monolayer for 2 h, then the culture medium was replaced with DMEM containing 2% FBS and supplemented with 1.25% carboxymethyl-cellulose (Millipore, Cat. 9004-32-4). At 72 hpi, Vero cell monolayers were fixed with 4% formaldehyde in phosphate-buffered saline (PBS; Gibco, Cat. 10010500BT) and permeabilized by incubation with 0.5% Triton X-100 (Solarbio, Cat. T8200) in the balanced salt solution. The cells were then stained with a mouse monoclonal antibody against SFTSV NP (0.75 µg/mL) (16), and an anti-mouse horseradish peroxidase-conjugated secondary antibody (1:1,000 dilution).

### Immunoblotting analysis

The cells and tissues were lysed with RIPA lysis buffer (Beyotime, Cat. P0013B) supplemented with 1% PMSF (Thermofisher, Cat. 1861280) and a phosphatase inhibitor mixture (Applygen, Cat. P1260) on ice. The protein concentration in the cell lysates was determined using a BCA Protein Quantification Kit. Prior to immunoblotting, the protein samples were boiled with 5× SDS sample buffer at 100°C for 5 min. Subsequently, the protein lysates were separated by SDS-polyacrylamide gel electrophoresis (PAGE) and transferred onto polyvinylidene difluoride (PVDF) membranes (Millipore, Cat. IPVH00010). After blocking with 5% skim milk (BD Difco, Cat. 232100) in Tris-buffered saline containing 0.05% Tween 20 (TBST) for 1 h at room temperature, the membranes were incubated overnight at 4°C with the indicated primary antibodies, followed by horseradish peroxidase-conjugated secondary antibodies. The sources of the aforementioned antibodies are listed in Table S6.

### Evaluation of the effect of glucose on SFTSV infection

Cells pre-seeded in a 24-well plate (1 × 10^5^ cells per well) were cultured in glucose-free DMEM medium (Gibco, Cat. 11966025) supplemented with 10% fetal bovine serum, 1% penicillin-streptomycin, 1 mM sodium pyruvate (Gibco, Cat. 11360070), and different final concentrations of glucose (Sigma, Cat. SLCC4951) for 12 h. Mannitol (Sigma, Cat. M4125) was used as the osmotic control for glucose levels. The cells were then infected with SFTSV at an MOI of 0.1 for 24 h. Intracellular SFTSV RNA levels and NP levels, as well as viral titers in the supernatant, were analyzed using the methods described above.

### Measurement of cell viability

Cytotoxicity was measured using the Cell Counting Assay Kit-8 (Applygen, Cat. E1008). Cells were seeded at a density of 1 × 10^6^ cells per mL in a 96-well plate and treated with metformin for 24 h. After that, each well was supplemented with 10 μL of CCK-8 solution and incubated for an additional 1–4 h at 37°C. The absorbance at 450 nm was measured byusing a microplate reader.

### Time-of-addition assay

For the time-of-addition assay, Huh7 cells were infected with SFTSV at an MOI of 1. Specifically, in the pre-group, cells were pre-treated with metformin (5 mM; Selleck, Cat. S1950) for 1 h, and incubated with SFTSV for 2 h, before removing both metformin and virus. In the post-group, cells were infected with SFTSV for 2 h, and metformin was added after removing the virus. In the whole group, cells were treated with metformin from 1 h before infection until 24 hpi. Infected cells were collected at 24 hpi and subjected to RT-qPCR and immunoblotting analysis.

### RNA interference knockdown

Huh7 cells and HUVECs pre-seeded in 24-well plates (1 × 10^5^ cells per well) were transfected with small interfering RNAs (siRNAs) using Lipofectamine RNAiMAX (Invitrogen, Cat. 13778150). Briefly, 10 pmol siRNA and 1 µL RNAiMAX were incubated at room temperature for 5 min before adding the siRNA–lipid complex to the cells. Scrambled siRNA was included as a negative control in parallel experiments. The efficiency of interference was determined by immunoblotting analysis after 72 h of transfection. The specific siRNA for AMPKα1/2 was purchased from Santa Cruz Biotechnology (Cat. sc-45312). The negative control siRNA: 5′-UUCUCCGAACGUGUCACGUTT-3′ was synthesized by GenePharma Co., Ltd (Jiangsu, China).

### Animal study

The mice were kept in a humidity-controlled (22 ± 2°C, 50% ± 10%) specific-pathogen-free (SPF) environment with 12 h light/12 h dark, and had *ad libitum* access to food and water in the State Key Laboratory of Pathogens and Biosecurity (Beijing, China).

C57BL/6J mice were obtained from Vital River Laboratories (Beijing, China). Leptin receptor-deficient C57BLKS/J-*Lepr^db^/Lepr^db^* mice (BKS-db/db) and non-diabetic mice (BKS-db/m) were obtained from GemPharmatech Co., Ltd (Jiangsu, China). To create non-genetic mouse models of STZ/HFD-induced T2D diabetes, 4-week-old female C57BL/6J mice were fed either an HFD (60 kcal% from fat, Research Diets, #D12492) or a control diet (10 kcal% from fat, Research Diets, #D12450J) for 4 weeks. After fasting for 4 hours, they received intraperitoneal injections of low-dose STZ (Med Chem Express, Cat. HY-13753) dissolved in 0.1 M sodium citrate buffer (pH 4.5, Solarbio, Cat. C1013) (40 mg/kg/day for 4 days), aiming to induce partial insulin deficiency. The mice continued to be maintained on an HFD or control diets for another week. Blood samples were collected from the tip of the tail to determine serum glucose levels. Mice with glucose concentrations over 200 mg/dL within the T2D groups qualified as suitable animal models (62).

To investigate the impact of SFTSV infection on blood glucose levels, 6-week-old female C57BL/6J mice were intraperitoneally injected with 100 µL of virus solution (2 × 10^6^ plaque-forming units [PFU]/mL). At 1 and 3 dpi, blood samples were collected for the detection of blood glucose and enzyme activities of PEPCK and G6Pase. Moreover, liver samples were collected for the detection of viral loads and enzyme activities of PEPCK and G6Pase.

To investigate the effect of pre-existing diabetes on the prognosis of SFTSV infection, female BKS-db/db and BKS-db/m mice were intraperitoneally injected with 100 µL of virus solution (2 × 10^6^ PFU/mL). At 3 dpi, blood samples were collected for the detection of blood glucose and virus loads. Meanwhile, lung, spleen, and liver samples were collected for the detection of virus loads and histopathological analysis. In addition, the association between blood glucose levels and viral loads was also investigated in the HFD/STZ-induced T2D mouse model as described above.

To evaluate the antiviral effect of metformin, a lethal mouse model was established using an anti-interferon α/β receptor subunit 1 (IFNAR1) blocking antibody (Bio X Cell, Cat. BE0241) pretreatment (16). Briefly, 6-week-old female BKS-db/db mice were treated with anti-IFNAR1 IgG (300 µg per mouse) by intraperitoneal injection 1 day before infection. Mice were intraperitoneally injected with 100 µL of virus solution (4 × 10^5^ PFU/mL). The metformin was dissolved in saline and administered by a stomach probe at a dose of 300 mg/kg per day. Metformin therapy lasted for 10 days, including 5 days prior to SFTSV infection and 5 days during viral infection. All mice were monitored daily for signs of disease (hunched posture, ruffled fur, decreased activity, and response to stimuli) and body weight for 14 days. At 5 dpi, blood, lung, spleen, and liver samples were collected for quantification of viral loads. The tissue samples were also subjected to histopathological analysis and immunoblotting analysis.

### Statistics and reproducibility

Continuous variables were described as means and standard deviations (s.d.) and compared using Student’s *t*-test or a non-parametric test between two groups, using one-way analysis of variance (ANOVA) followed by Tukey’s multiple-comparison test among multiple groups, or using two-way ANOVA for examining the impact of two independent variables. Pearson’s correlation coefficient was calculated to assess the relationship between blood glucose levels and viral loads. We analyzed the time-to-event data for treatment effect analysis using the Kaplan-Meier method and the log-rank test. Multivariable Cox regression models, which adjusted for age, sex, onset-to-admission interval, and pre-existing comorbidities, were employed. All statistical analyses were performed using R software version 4.2.2.

## Data Availability

All data associated with this study are present in the paper or the supplemental material.

## SUPPLEMENTAL MATERIAL

Supplemental material mbio.00634-25-s0001.docx Supplemental figures and tables.

ASM does not own the copyrights to Supplemental Material that may be linked to, or accessed through, an article. The authors have granted ASM a non-exclusive, world-wide license to publish the Supplemental Material files. Please contact the corresponding author directly for reuse.
