# Supplementary material for: Metformin as antiviral therapy protects hyperglycemic and diabetic patients
Source: mBio. 2025 May 20;16(6):e00634-25. doi: 10.1128/mbio.00634-25 (PMC12153287; doi:10.1128/mbio.00634-25)
Supplement: Supplemental material — Supplemental figures and tables. [file mbio.00634-25-s0001.docx]

**Supplementary Figures and Tables**

**Supplementary Figure 1. Sex-stratified analysis of the impact of hyperglycemia on mortality and viremia in SFTSV-infected individuals without underlying diabetes.**


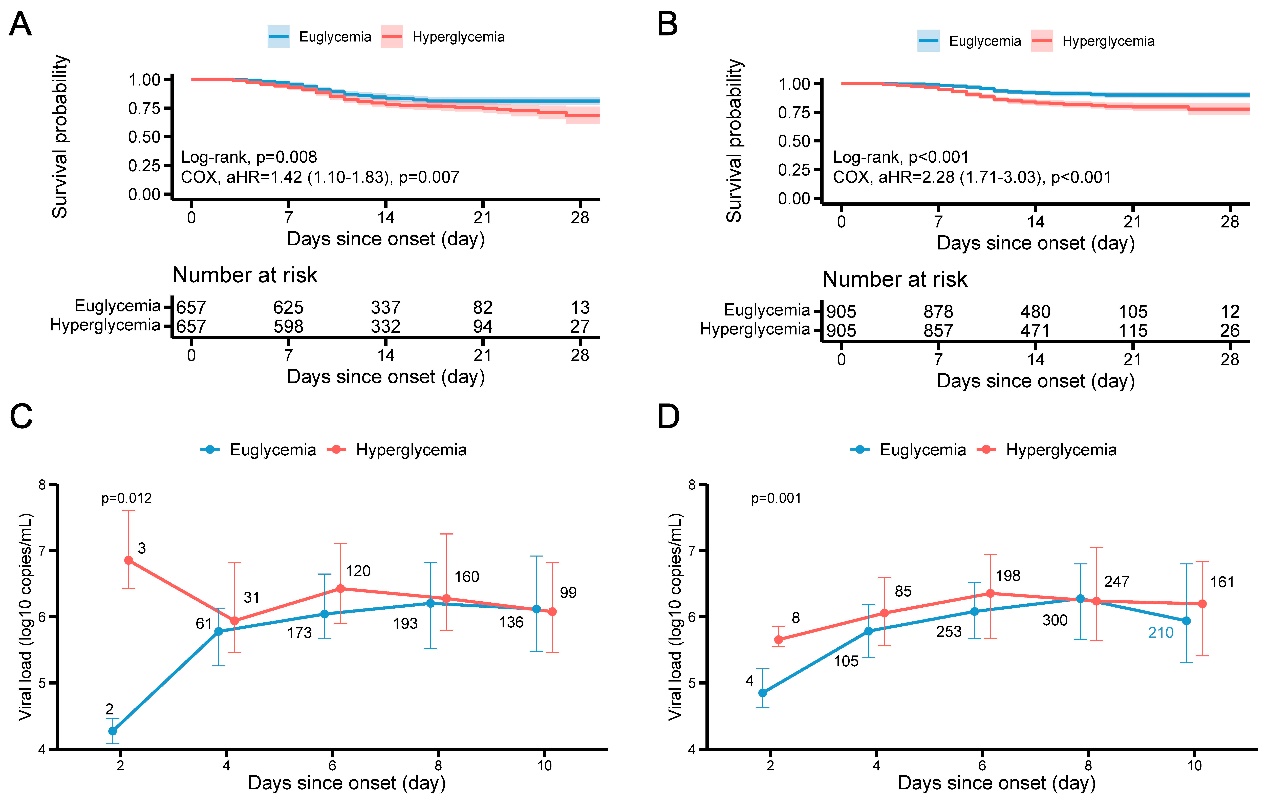


**A**, **B** Analysis of hyperglycemia on survival probability of SFTS patients in males (**A**) and females (**B**). The Kaplan-Meier method was used to analyze time-to-event data. Adjusted HR and 95% CI were conducted by a multivariable COX regression with adjustment for age, onset-to-admission interval, and pre-existing comorbidities.

**C**, **D** Dynamic profiles of SFTSV viral loads between hyperglycemia and euglycemia group in males (**C**) and females (**D**). Data were presented as median and interquartile range. GEE model was performed to consider the effect from age, onset-to-admission interval. The number of SFTS patients included for analysis at each time point were added to line graphs.

**Supplementary Figure 2. SFTSV infection triggers gluconeogenesis in HepG2 cells.**


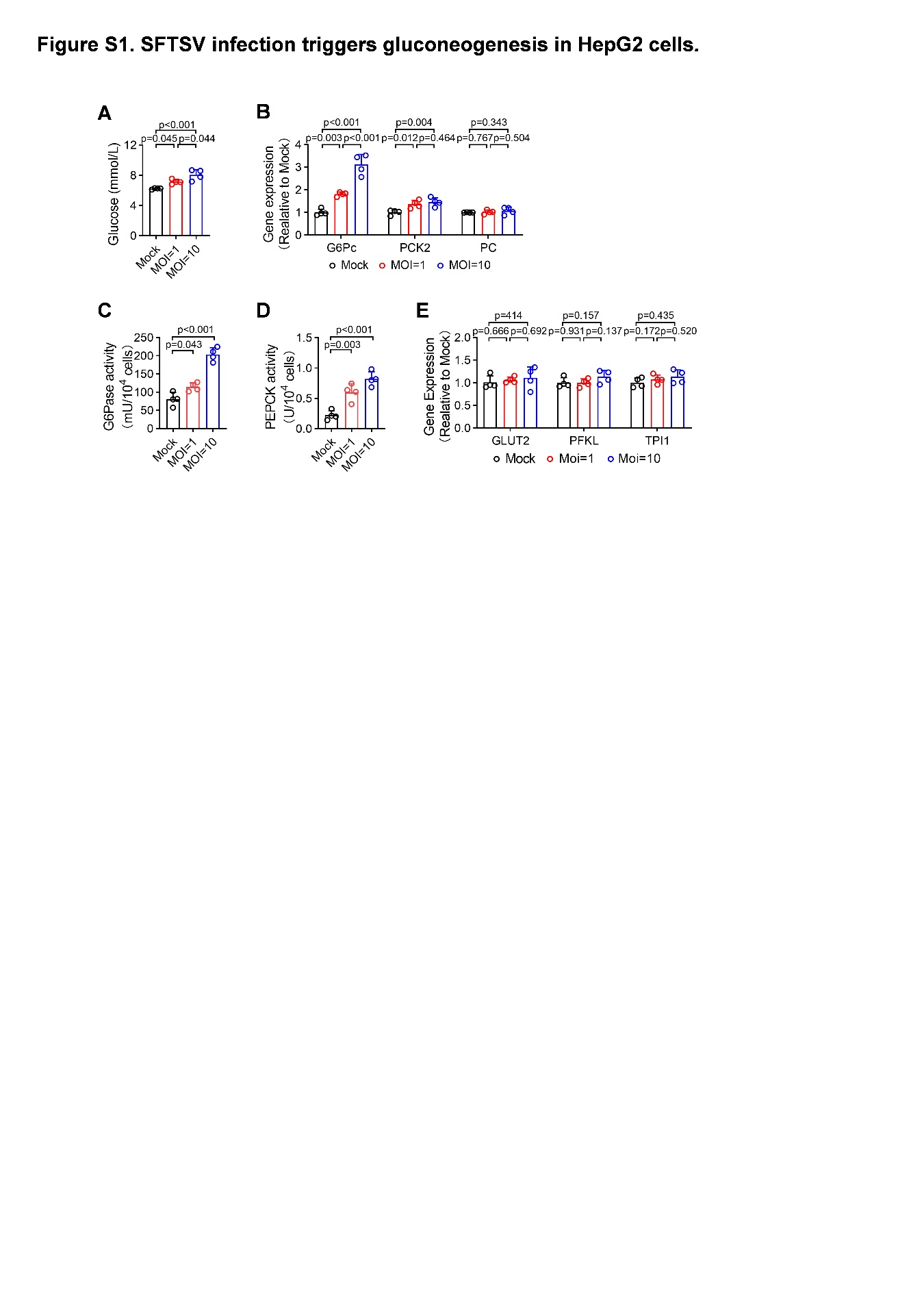


**A** Glucose level was measured in supernatant of SFTSV-infected HepG2 cells at 48 hours post-infection (hpi); n = 4 biologically independent samples.

**B**-**E** The mRNA levels of gluconeogenesis genes, including *G6Pc*, *PCK2*, and *PC*, were measured in mock- or SFTSV-infected HepG2 cells at 48 hpi; n = 4 biologically independent samples.

**C**, **D** G6pase activity (**C**) and PEPCK activity (**D**) were measured in mock- or SFTSV-infected HepG2 cells at 48 hpi; n = 4 biologically independent samples.

**E** The mRNA levels of glycolysis genes, including *GLUT2*, *PFKL,* and *TPI1*, were measured in mock- or SFTSV-infected HepG2 cells at 48 hpi; n = 4 biologically independent samples.

Data were presented as mean ± s.d. The two-sided *p* values were examined using One-way ANOVA followed by Tukey’s multiple comparisons test for comparison of continuous variables among multiple groups (**A**-**E**).

**Supplementary Figure 3. High glucose increases the susceptibility of HepG2 cells and HUVECs to SFTSV infection.**


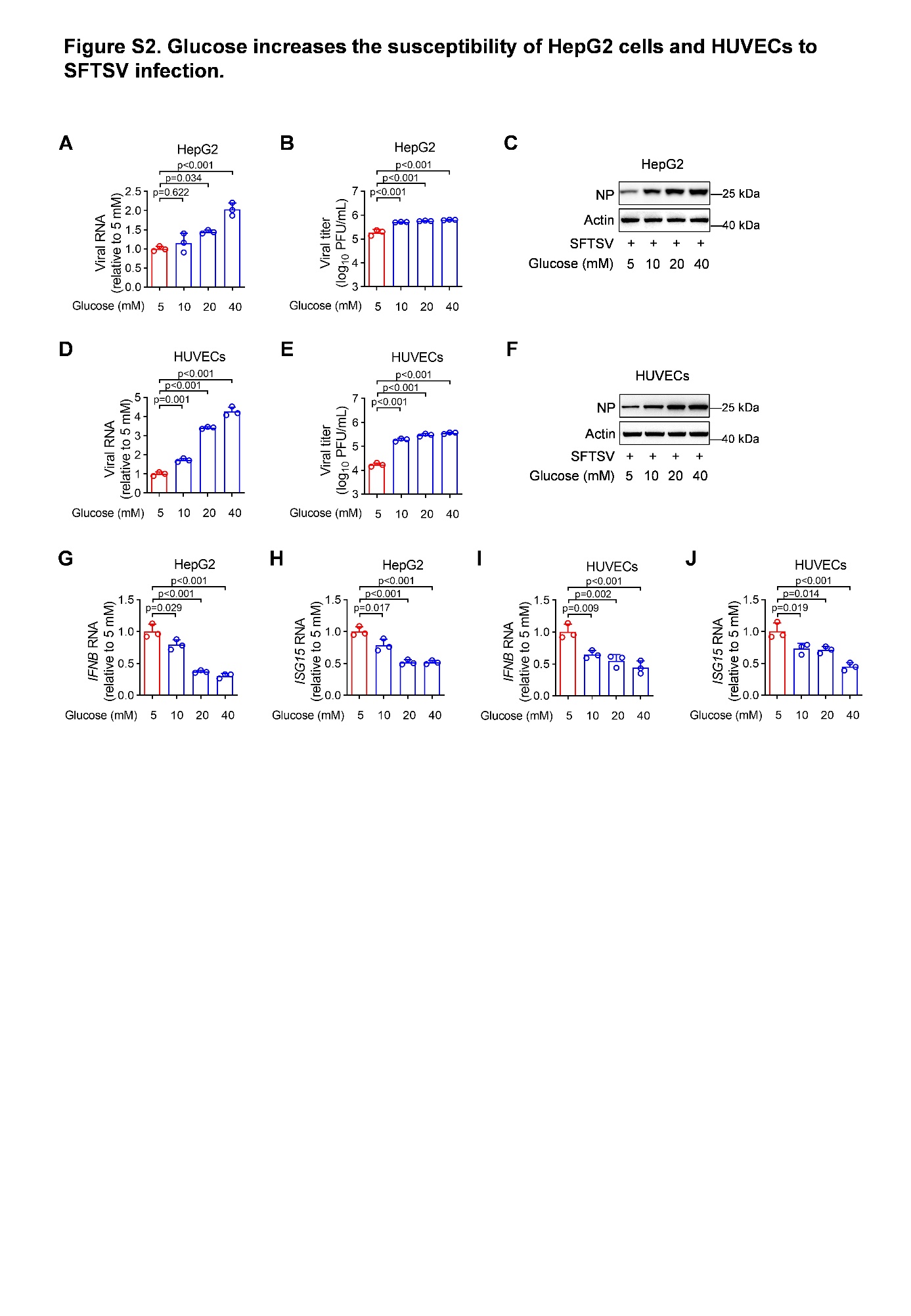


**A**-**C** Intracellular SFTSV RNA levels (**A**), supernatant viral titers (**B**), and intracellular NP levels (**C**), were measured in HepG2 cells infected with SFTSV (MOI = 0.1) and cultured with DMEM containing indicated glucose concentrations at 24 hours post-infection (hpi); n = 3 biologically independent samples.

**D**-**F** Intracellular SFTSV RNA levels (**D**), supernatant viral titers (**E**), and intracellular NP levels (**F**), were measured in HUVECs infected with SFTSV (MOI = 0.1) and cultured with DMEM containing indicated glucose concentrations at 24 hpi; n = 3 biologically independent samples.

Data were presented as mean ± s.d. The two-sided *p* values were examined using One-way ANOVA followed by Tukey’s multiple comparisons test for comparison of continuous variables among multiple groups (**A**, **B**, **D**, **E**, **G**-**J**). The presented images are representative of three independent experiments in immunoblotting analysis (**C**, **F**).

**Supplementary Figure 4.** **Quantification and normalization analysis for immunoblotting of whole-cell extracts from SFTSV-infected Huh7 cells.**


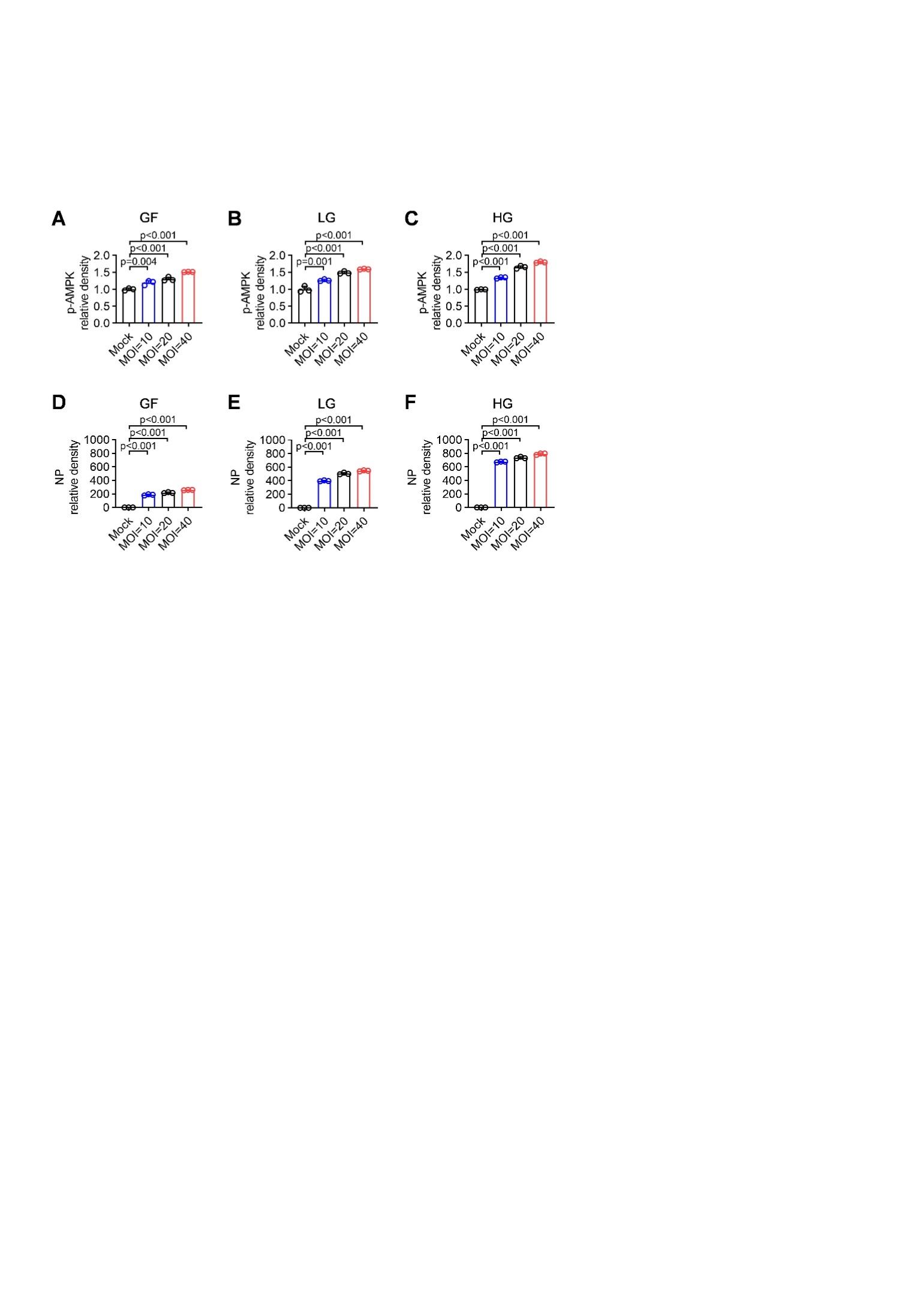


Whole-cell extracts (WCEs) from SFTSV-infected Huh7 cells cultured in glucose-free (0 mM glucose, GF), low-glucose (5mM glucose, LG) or high-glucose (20 mM glucose, HG) DMEM were analyzed by immunoblotting analysis using indicated antibodies at 24 hours post-infection. The relative density of p-AMPK (**A-C**) and NP (**D-F**) was compared among groups. Data were presented as mean ± s.d. The two-sided *p* values were examined using One-way ANOVA followed by Tukey’s multiple for comparison of variables among groups.

**Supplementary Figure 5. Effect of high glucose on the type I interferon response in SFTSV-infected cells.**


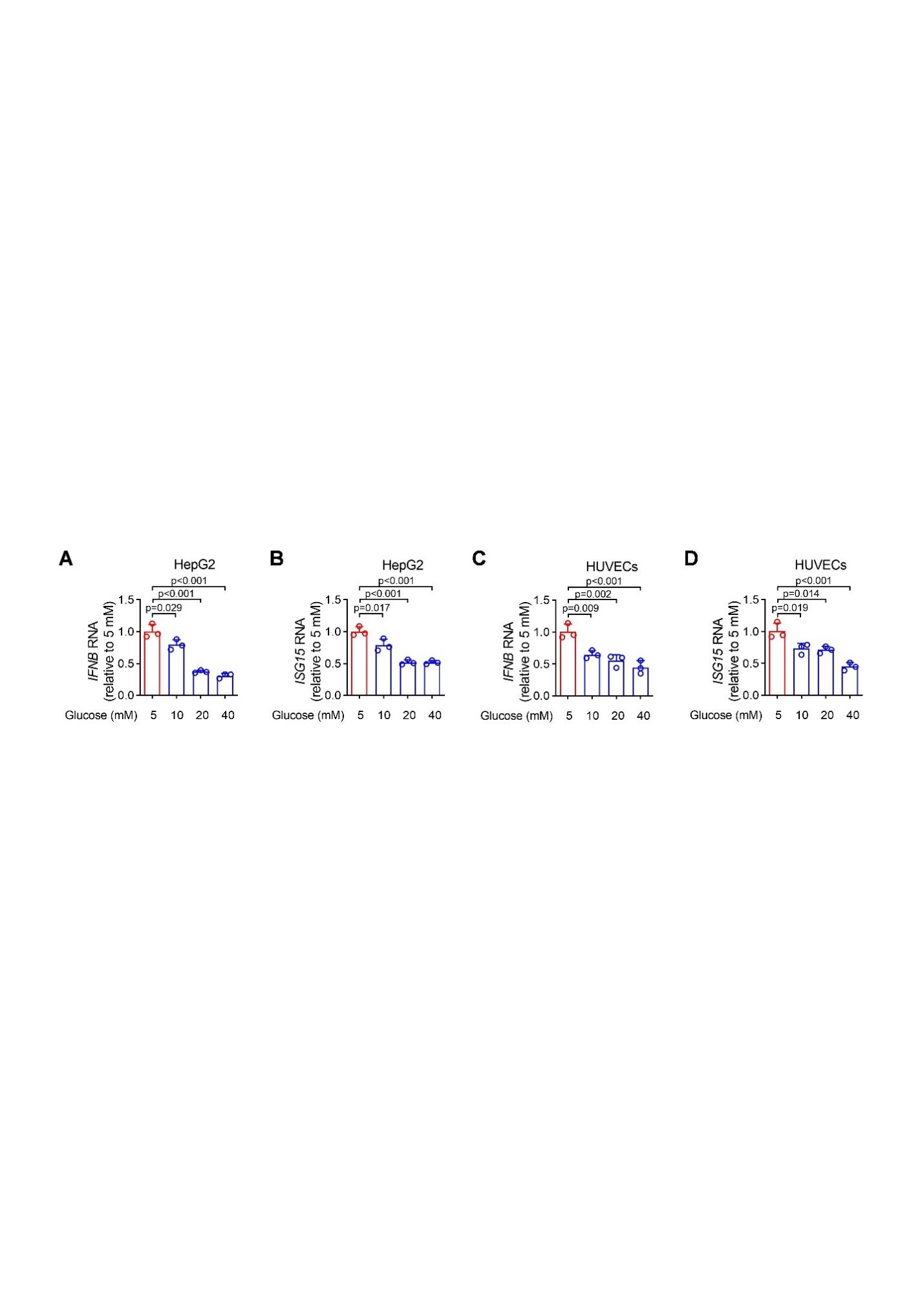


**A**-**D** The mRNA levels of *IFNB* and *ISG15* were measured in HepG2 cells (**A,** **B**) or HUVECs (**C**, **D**) infected with SFTSV (MOI = 0.1) and cultured with DMEM containing indicated glucose concentrations at 24 hpi; n = 3 biologically independent samples. Data were presented as mean ± s.d. The two-sided *p* values were examined using One-way ANOVA followed by Tukey’s multiple comparisons test for comparison of continuous variables among multiple groups.

**Supplementary Figure 6. Sex-stratified analysis of the impact of underlying diabetes on mortality and viremia in SFTSV-infected individuals.**


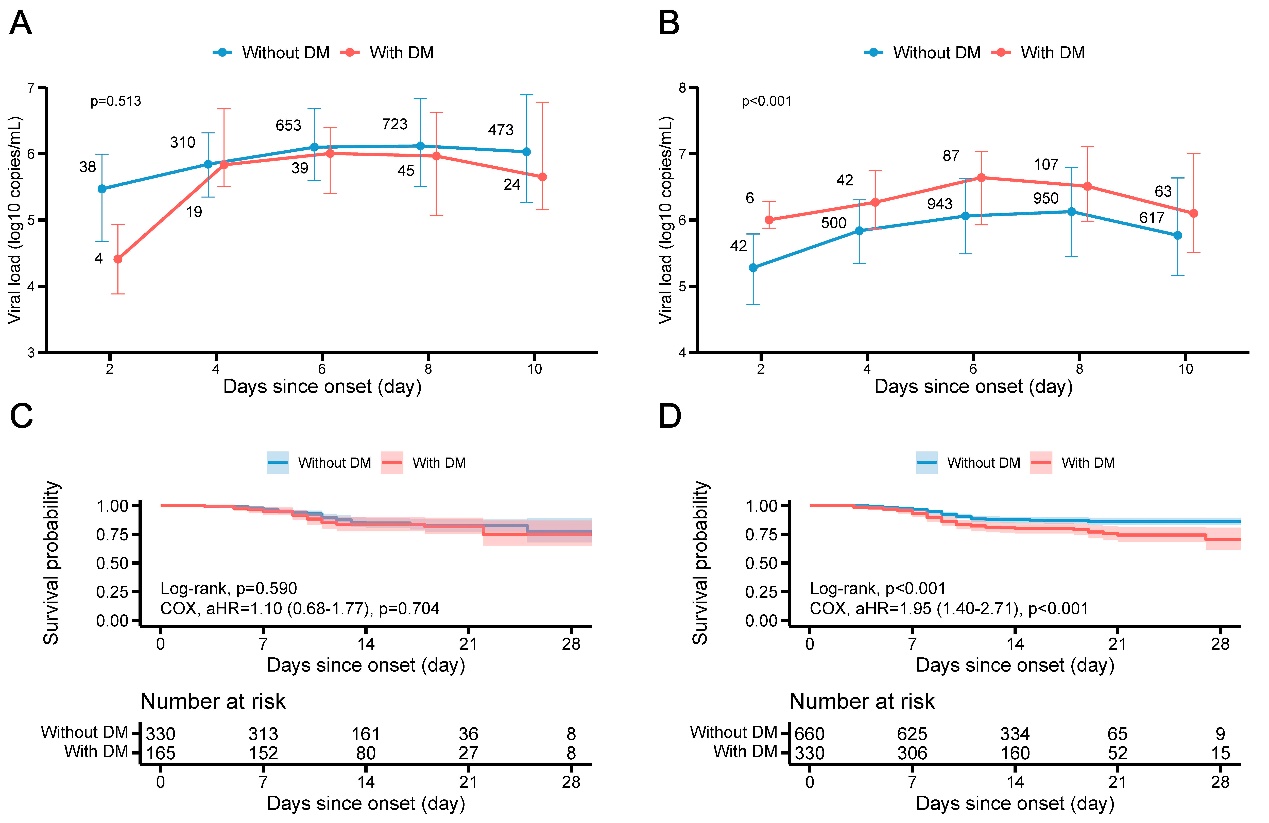


**A**, **B** Dynamic profiles of SFTSV viral loads between patients with diabetes and those without diabetes in males (**A**) and females (**B**). Data were presented as median and interquartile range. GEE model was performed to consider the effect from age, onset-to-admission interval. The number of SFTS patients included for analysis at each time point were added to line graphs.

**C**, **D** Analysis of underlying diabetes on survival probability in males (**C**) and females (**D**). The Kaplan-Meier method was used to analyze time-to-event data. Adjusted HR and 95% CI were conducted by a multivariable COX regression adjusted for age, onset-to-admission interval, and pre-existing comorbidities.

**Supplementary Figure 7. Clinical effects of metformin in treating SFTS patients with underlying diabetes and lowering glucose.**


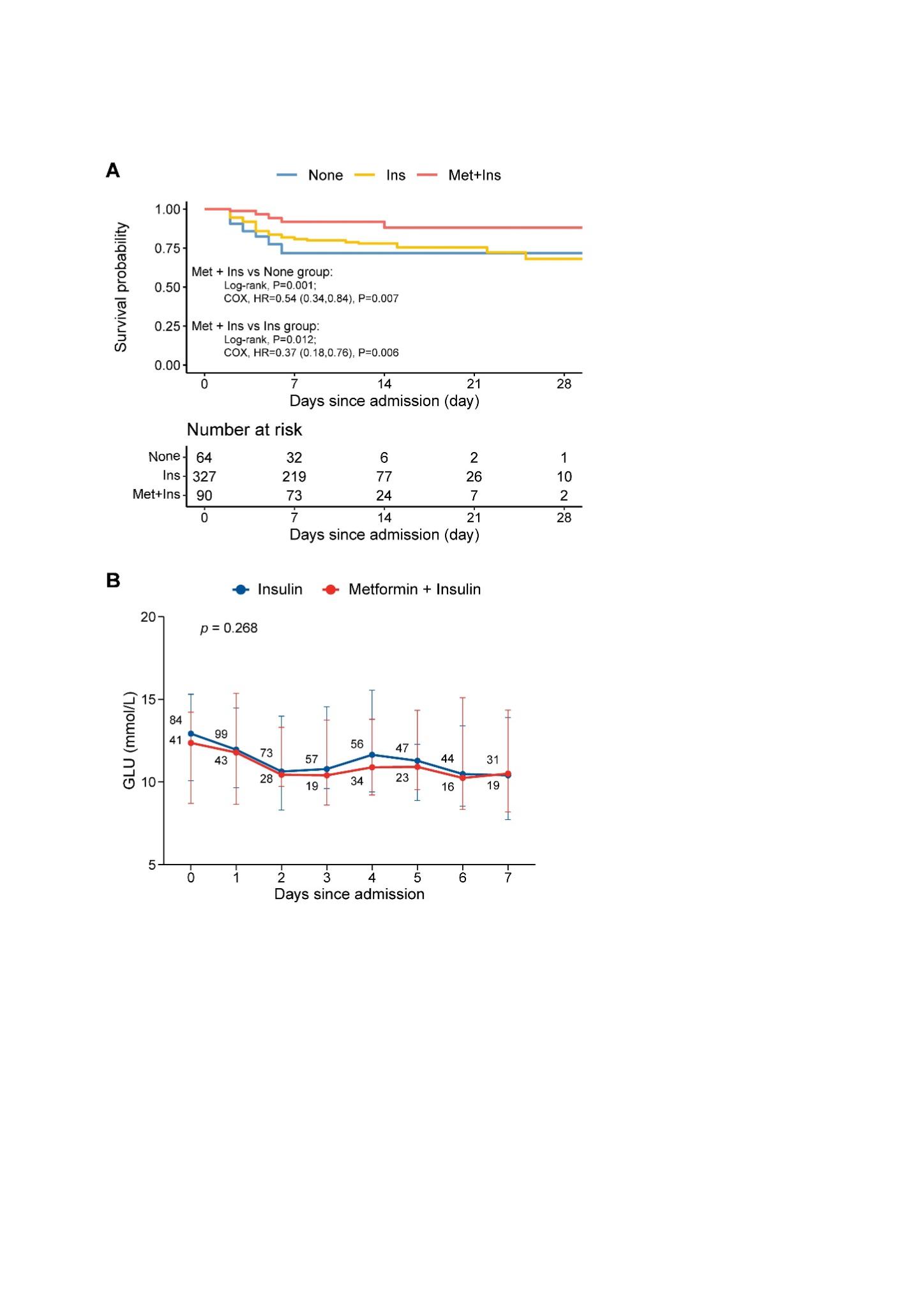


**A** Analysis of treatment of glucose-lowering drugs on survival probability of SFTS patients. The Kaplan-Meier method was used to analyze time-to-event data. Adjusted HR and 95% CI was conducted by a multivariable COX regression adjusted for age, sex, onset-to-admission interval, and preexisting comorbidity.

**B** Dynamic profiles of blood glucose levels between patients receiving combined metformin and insulin and those receiving insulin treatment alone. Data were presented as median and interquartile range. GEE model was performed to consider the effect from age, sex, onset-to-admission interval. The number of SFTS patients included for analysis at each time point were added to line graphs.

**Supplementary Figure 8. Sex-stratified analysis of** **clinical efficacy of metformin in treating SFTS patients with underlying diabetes.**


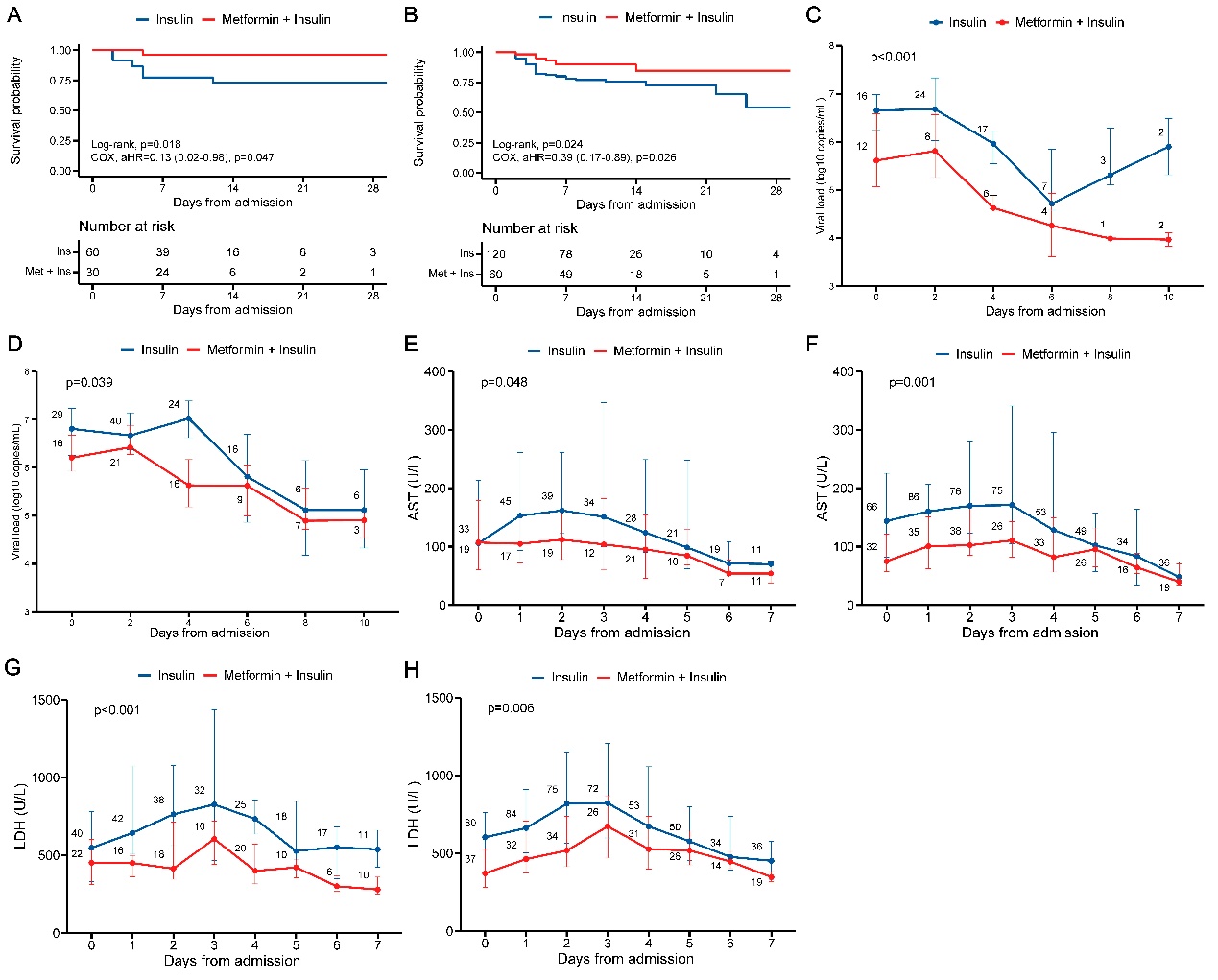


**A**, **B** Analysis of metformin treatment on survival probability of SFTS patients in males (**A**) and females (**B**). The Kaplan-Meier method was used to analyze time-to-event data. Adjusted HR and 95% CI was conducted by a multivariable COX regression adjusted for age, onset-to-admission interval, and pre-existing comorbidity.

**C-J** Dynamic profiles of SFTSV viral load (**C, D**), AST (**E, F**), and LDH (**G, H**) between patients receiving combined metformin and insulin treatment and those receiving insulin treatment alone in males (**C, E, G**) and females (**D, F, H**). Data were presented as median and interquartile range. GEE model was performed to consider the effect from age, onset-to-admission interval. The number of SFTS patients included for analysis at each time point were added to line graphs.

**Supplementary Figure 9. Clinical effects of metformin in treating SFTS patients with hyperglycemia but without DM.**


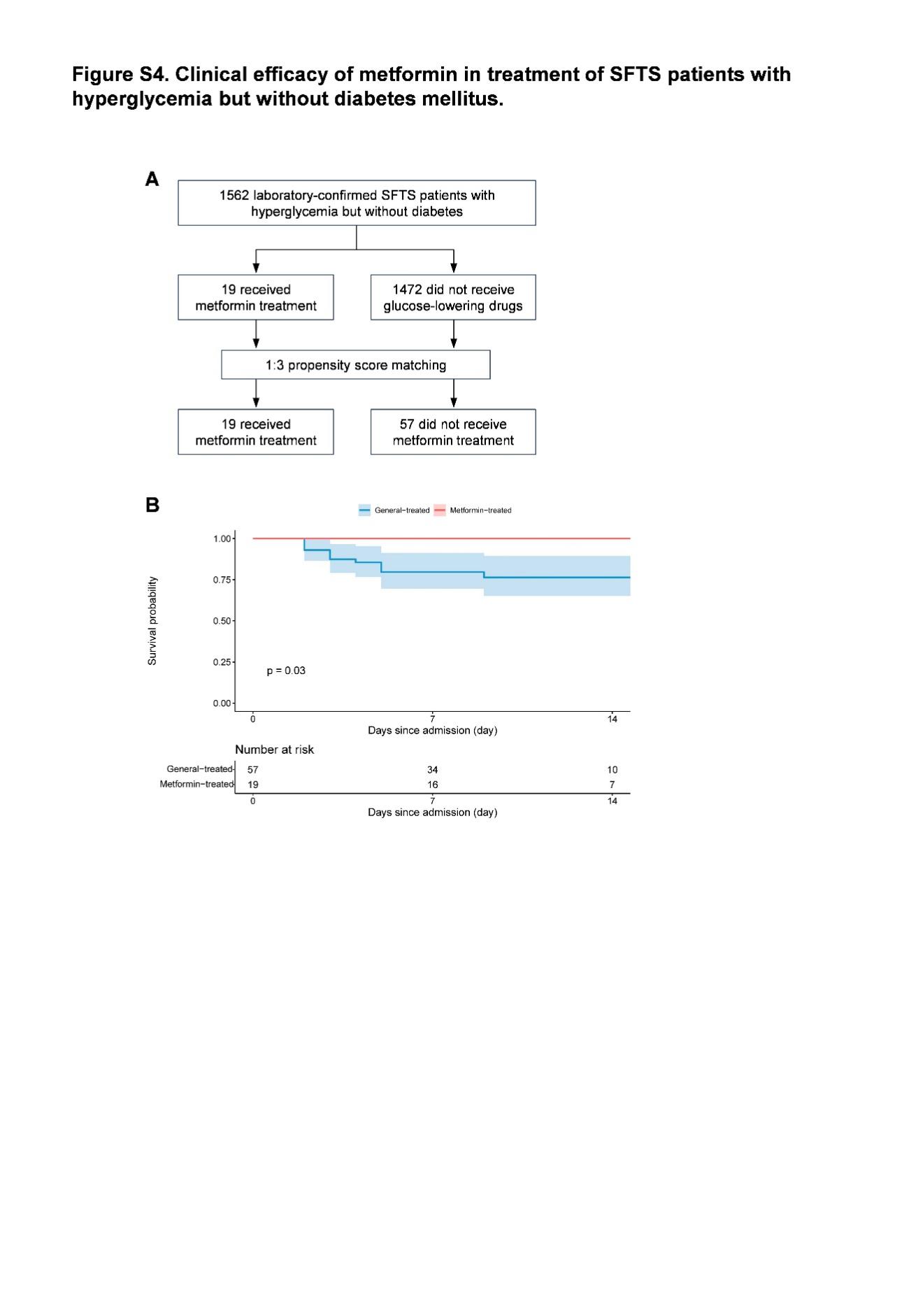


**A** Flow chart of the recruitment and grouping process for SFTS patients. Among 1,562 laboratory-confirmed SFTS patient with hyperglycemia but without diabetes recruited, 19 patients receiving metformin treatment and 59 patients without metformin treatment were included for analysis.

**B** Analysis of metformin treatment on survival probability of SFTS patients. The Kaplan-Meier method was used to analyze time-to-event data.

**Supplementary Figure 10. Effect of metformin and insulin on SFTSV infection and cell viability.**


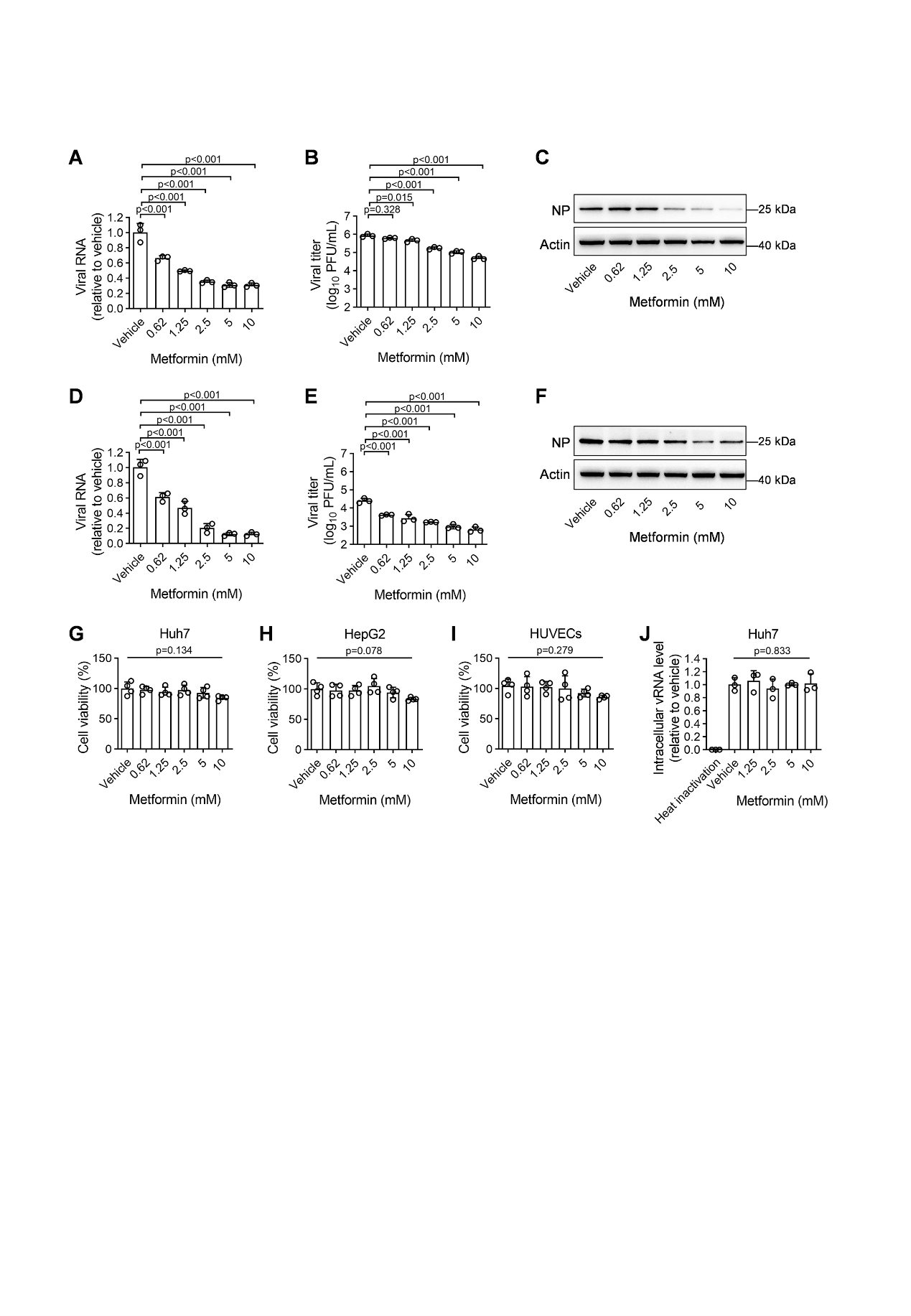


**A**-**F** Intracellular SFTSV RNA levels (**A**, **D**), supernatant viral titers (**B**, **E**), and intracellular NP levels (**C**, **F**), were measured in HepG2 cells (**A**-**C**) or HUVECs (**D**-**F**) infected with SFTSV (MOI = 1) and cultured with DMEM containing indicated concentrations of metformin at 24 hours post-infection (hpi); n = 3 biologically independent samples.

**G**-**I** Viability of metformin treated Huh7 cells (**G**), HepG2 cells (**H**), and HUVECs (**I**) was measured at 24 hours post treatment using CCK-8 assay; n = 4 biologically independent samples.

**J** SFTSV was incubated with indicated concentrations of metformin for 1 h at 37℃, and the mixture was then added to Huh7 cells. Intracellular SFTSV RNA level was measured at 24 hpi; n = 3 biologically independent samples.

Data were presented as mean ± s.d. The two-sided *p* values were examined using One-way ANOVA followed by Tukey’s multiple comparisons test for comparison of continuous variables among multiple groups (**A**, **B**, **D**, **E**). The presented images are representative of three independent experiments in immunoblotting analysis (**C**, **F**).

**Supplementary Figure 11. Effect analysis of metformin on the IFN-I response in SFTSV-infected cells.**


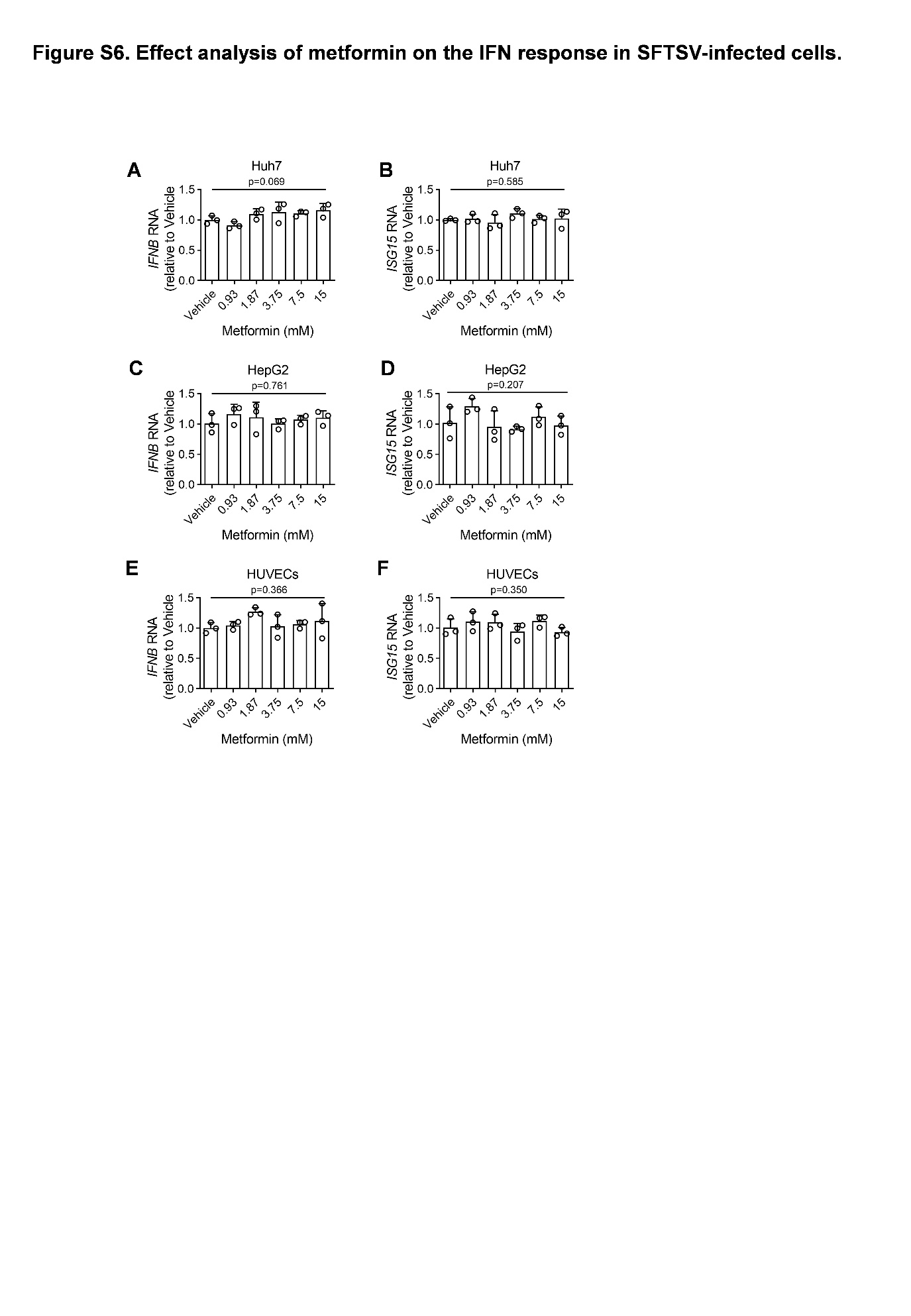


**A**-**F** The mRNA levels of *IFNB* (**A**, **C**, **E**) and *ISG15* (**B**, **D**, **F**) were measured in Huh7 cells (**A**, **B**), HepG2 cells (**C**, **D**), or HUVECs (**E**, **F**) infected with SFTSV (MOI = 0.1) and treated with indicated concentrations of metformin at 24 hours post-infection; n = 3 biologically independent samples.

Data were presented as mean ± s.d. The two-sided *p* values were examined using One-way ANOVA followed by Tukey’s multiple comparisons test for comparison of continuous variables among multiple groups.

**Supplementary Figure 12. Knockdown efficiency and cytotoxicity effect of siAMPK in Huh7 cells.**


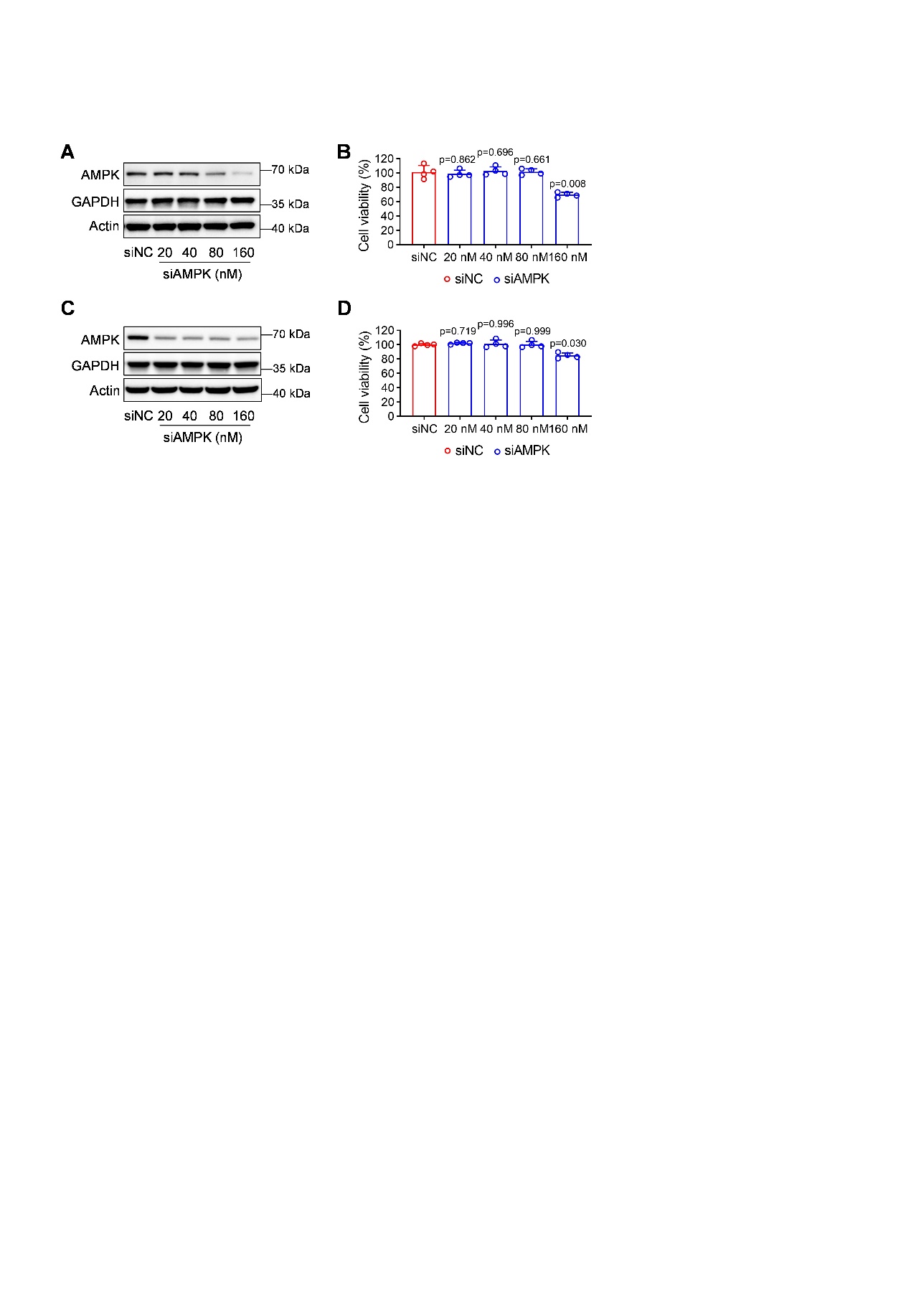


**A-D** Western blot analysis of AMPK expression levels in siAMPK-treated Huh7 cells (**A**) and HUVECs (**B**). Analysis of the viability of siAMPK-treated Huh7 cells (**C**) and HUVECs (**D**). Equal numbers of cells were plated, and viability was assessed over a 24-hour period using a cell counting kit-8; n = 3 biologically independent samples. Data were presented as mean ± s.d. The two-sided *p* values were examined using One-way ANOVA followed by Tukey’s multiple comparisons test for comparison of continuous variables among multiple groups.

**Supplementary Figure 13. Effects of AMPK knockdown on the type I response in SFTSV-infected cells treated with metformin.**


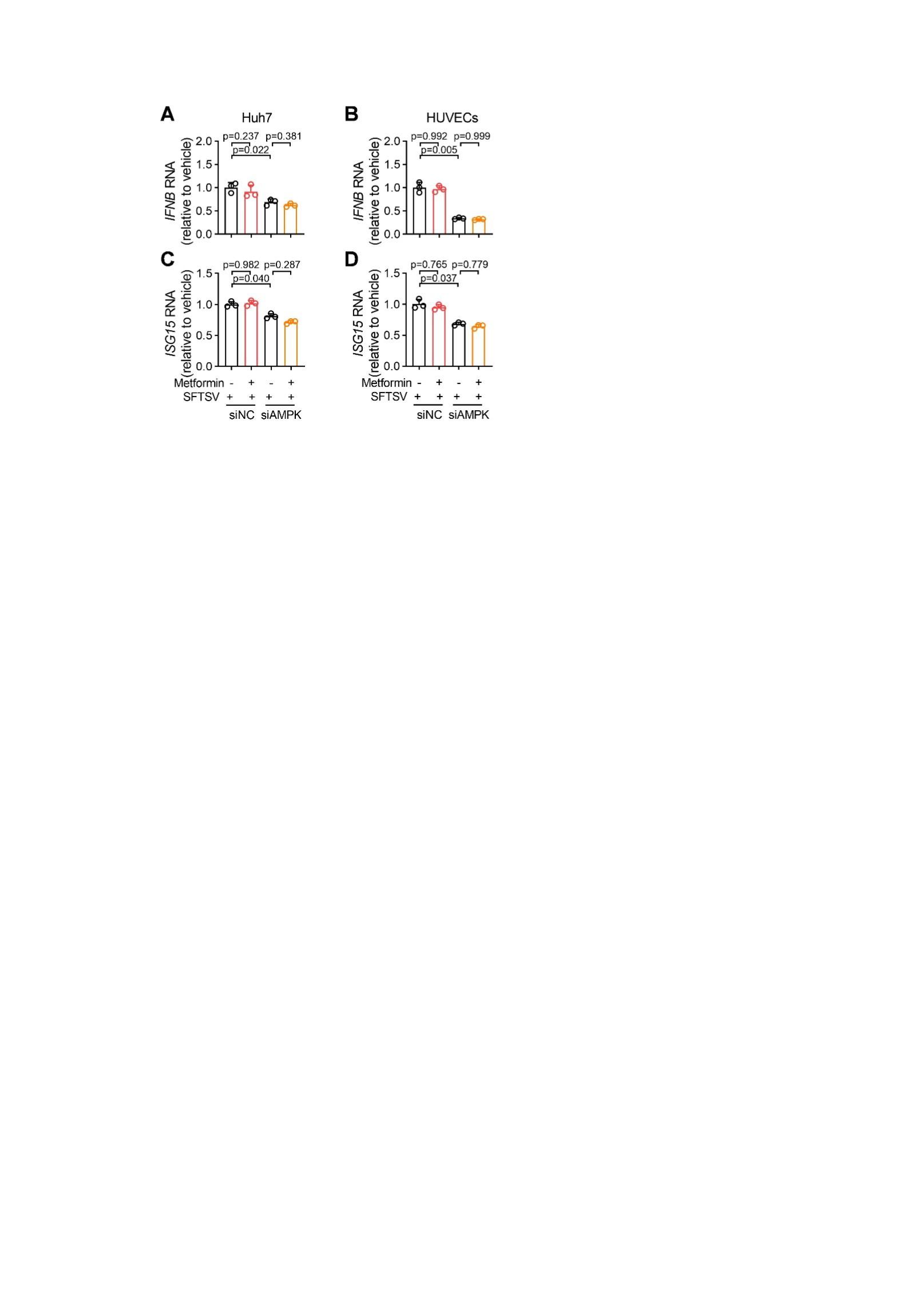


**A-D** The mRNA levels of *IFNB* (**A**, **B**) and *ISG15* (**C**, **D**) were measured in AMPK-knockdown Huh7 cells (**A**, **C**) or HUVECs (**B**, **D**) infected with SFTSV (MOI = 1) and treated with indicated concentrations of metformin at 24 hours post-infection; n = 3 biologically independent samples. Data were presented as mean ± s.d. The two-sided *p* values were examined using Two-way ANOVA multiple comparisons test for comparison of continuous variables among multiple groups.

**Supplementary Figure 14. Effect analysis of metformin on the AMPK/mTOR and autophagy pathways in SFTSV-infected HUVECs.**


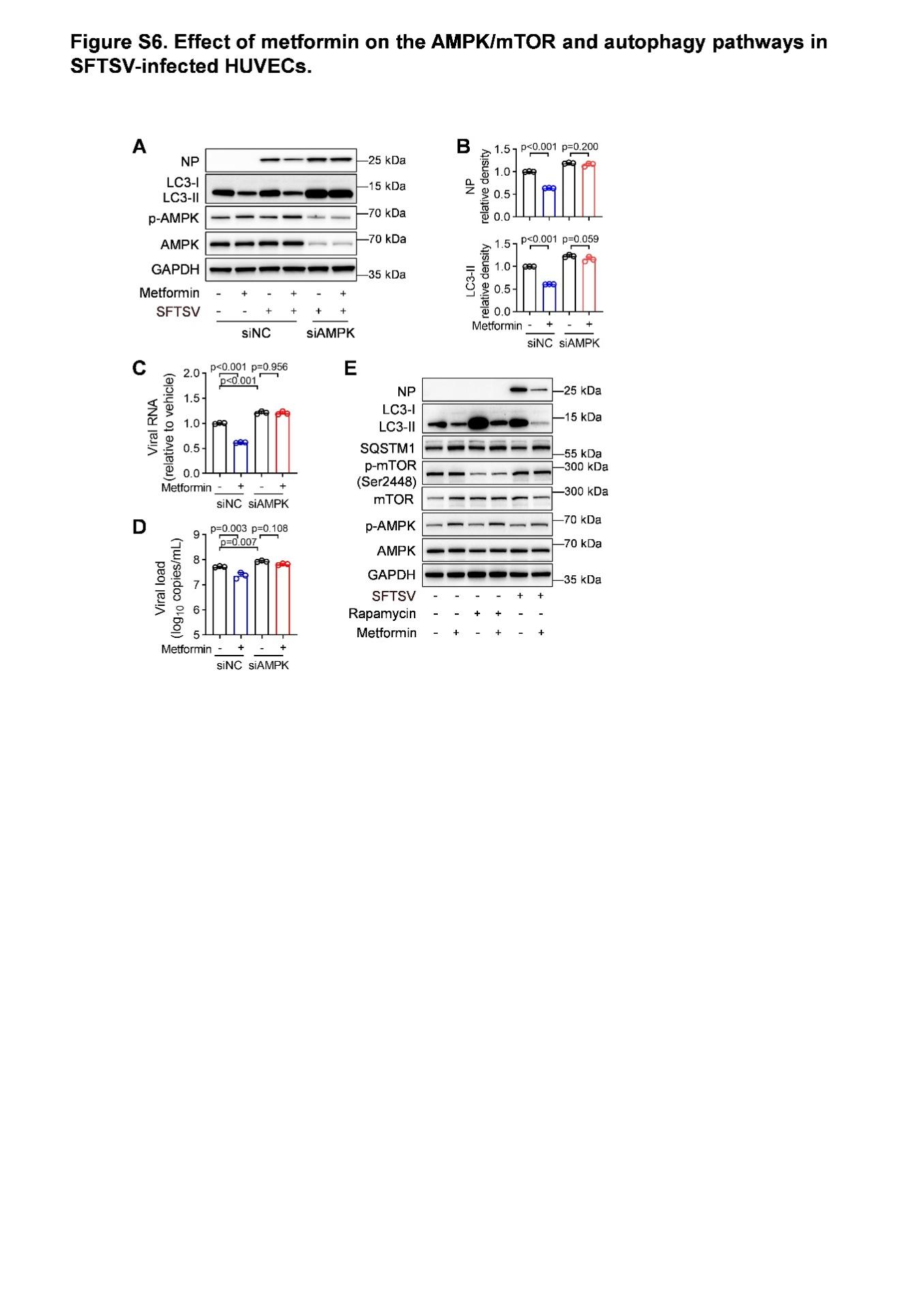


**A, B** AMPK-knockdown HUVECs (siAMPK concentration: 80 nM) were infected with SFTSV (MOI = 1) and treated with metformin. Indicated proteins in whole-cell extracts (WCEs) were examined by immunoblotting analysis (**A**), and the relative density of NP and LC3-II was compared among groups (**B**).

**C, D** Intracellular SFTSV RNA levels (**C**) and supernatant viral loads (**D**) were measured at 24 hours post infection; n = 3 biologically independent samples.

**E** WCEs from SFTSV-infected or rapamycin-treated HUVECs in presence or absence of metformin were analyzed by immunoblotting analysis using indicated antibodies.

Data were presented as mean ± s.d. The two-sided *p* values were examined using Tow-way ANOVA multiple comparison among groups (**B-D**). The presented images are representative of three independent experiments in immunoblotting analysis (**A**, **E**).

**Supplementary Figure 15. AMPK agonists AICAR suppresses SFTSV replication via AMPK-autophagy pathway in Huh7 cells.**


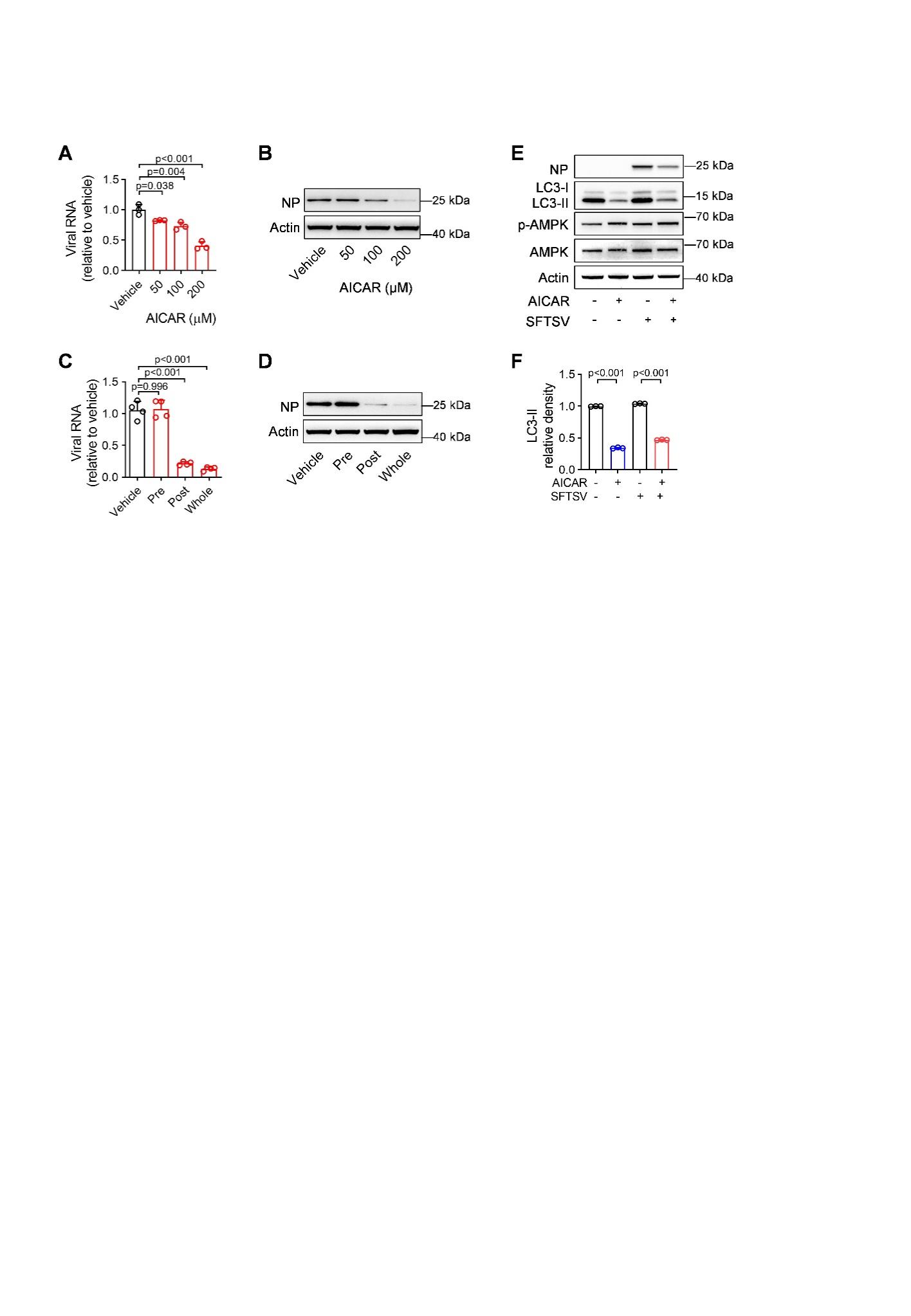


**A**, **B** Intracellular SFTSV RNA levels (**A**) and NP levels (**B**) were measured in Huh7 cells infected with SFTSV (MOI = 1) and cultured with indicated concentrations of AICAR at 24 hours post-infection (hpi); n = 3 biologically independent samples.

**C**, **D** Huh7 cells infected with SFTSV (MOI = 1) for 2 h were treated with vehicle (control) or AICAR in pre-infection, post-infection, or whole time. Intracellular SFTSV RNA levels (**C**) and NP levels (**D**) were measured at 24 hpi; n = 4 biologically independent samples.

**E, F** Whole-cell extracts from mock- or SFTSV-infected Huh7 cell treated with vehicle or AICAR were analyzed by immunoblotting analysis using indicated antibodies (**E**), and the relative density of LC3-II was compared among groups (**F**).

Data were presented as mean ± s.d. The two-sided *p* values were examined using One-way ANOVA followed by Tukey’s multiple comparisons test for comparison of continuous variables among multiple groups (**A**, **C**). The presented images are representative of three independent experiments in immunoblotting analysis (**B**, **D**-**F**).

**Supplementary Figure 16. Effect of insulin on SFTSV infection and cell viability.**


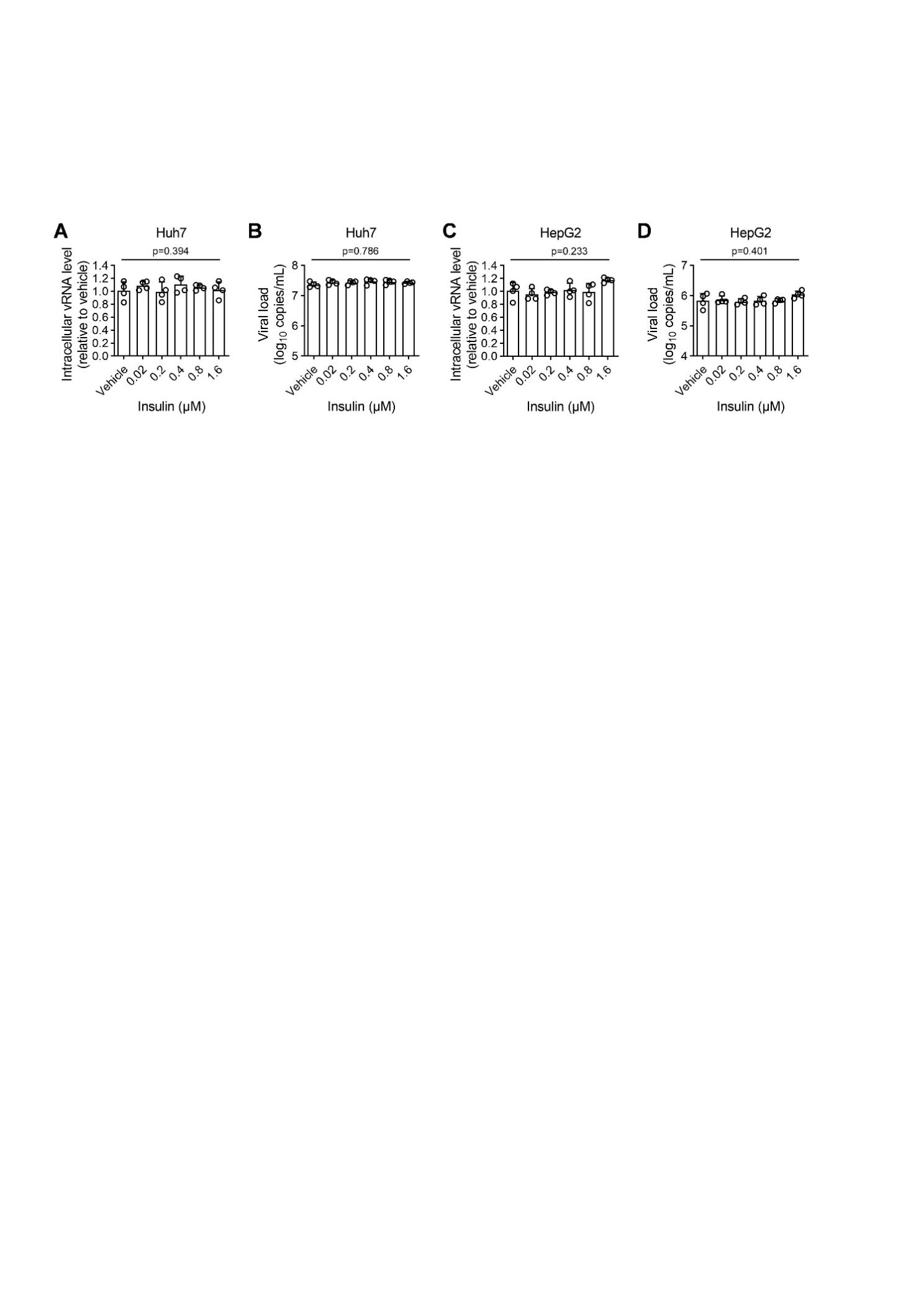


**A**-**D** Intracellular SFTSV RNA levels (**A**, **C**) and supernatant viral titers (**B**, **D**) were measured in Huh7 (**A**, **B**) and HepG2 cells (**C**, **D**) infected with SFTSV (MOI = 0.1) and cultured with DMEM containing indicated concentrations of insulin at 24 hpi; n = 4 biologically independent samples. Data were presented as mean ± s.d. The two-sided *p* values were examined using One-way ANOVA followed by Tukey’s multiple comparisons test for comparison of continuous variables among multiple groups.

**Supplementary Figure 17. Schematic illustration of the regulation of SFTSV by high glucose and metformin.**


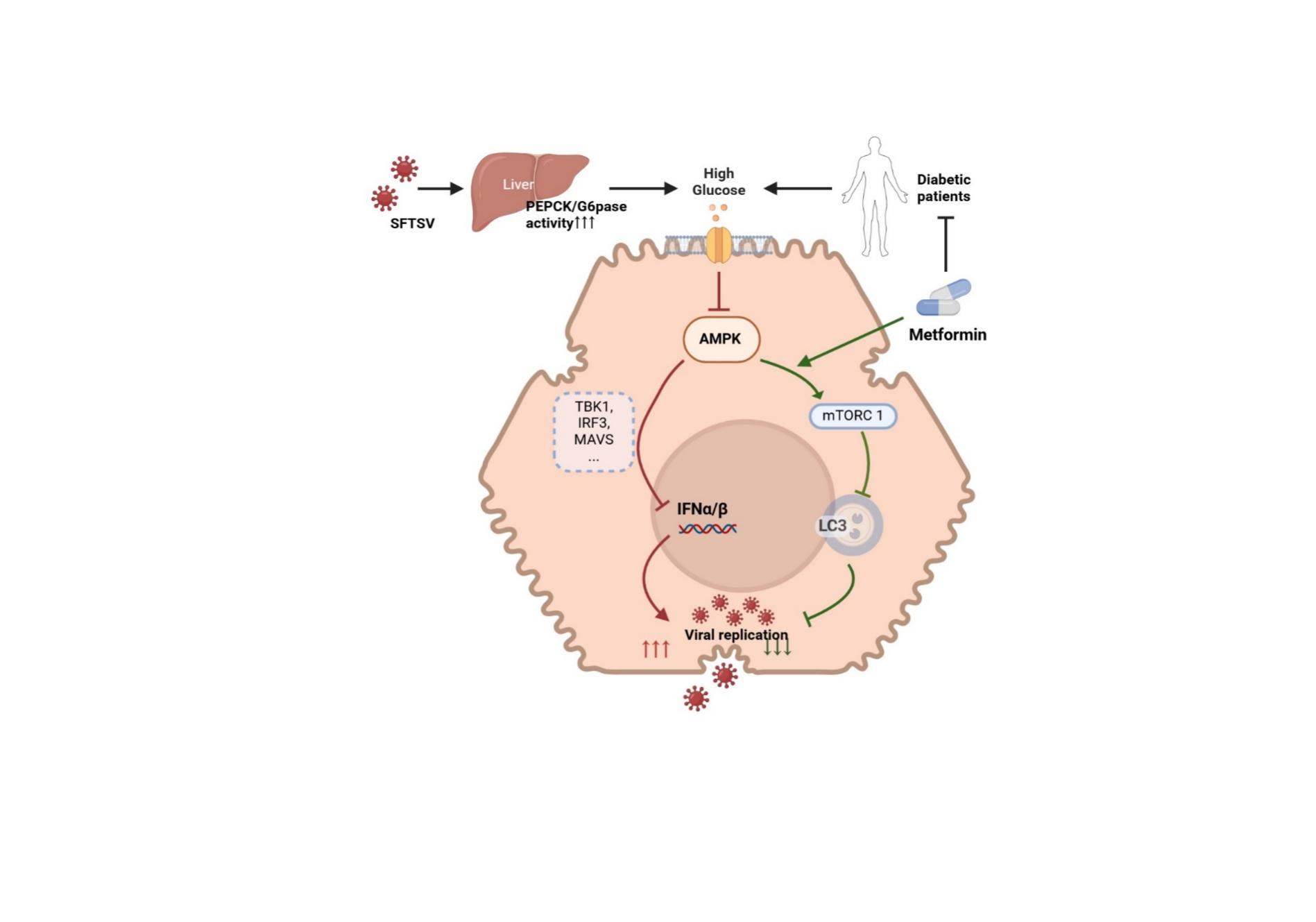


Upon SFTSV infection, the gene expression and enzymatic activities of PEPCK and G6Pase were significantly upregulated in liver cells, leading to the increase in glucose levels. High glucose induced by SFTSV infection or underlying diabetes inhibits AMPK activity and subsequent IFN-I signaling transduction, resulting in promoting SFTSV infection. Metformin, an antidiabetic agent, can suppress the replication of SFTSV, which is associated with its inhibition of autophagy via the AMPK-mTOR pathway.

**Supplementary Table 1. Comparisons of demographic features and clinical manifestations in SFTS patients with hyperglycemia and euglycemia.**

|  | Euglycemia (n=1562) | Hyperglycemia (n=1562) | *p* |  |
| --- | --- | --- | --- | --- |
| Age, years | 66 (57, 72) | 65.00 (57, 72) | 0.518 |  |
| Male | 653 (41.8) | 657 (42.1) | 0.913 |  |
| Delay from symptom onset to admission, days | 6 (4, 7) | 6 (5, 7) | 0.076 |  |
| Hypertension | 255 (16.3) | 273 (17.5) | 0.417 |  |
| Non-specific features |  |  |  |  |
| Fever | 1562 (100) | 1562 (100) | - |  |
| Chills | 449 (28.7) | 441 (28.2) | 0.781 |  |
| Headache | 262 (16.8) | 249 (15.9) | 0.562 |  |
| Dizzy | 323 (20.7) | 283 (18.1) | 0.078 |  |
| Feeble | 1321 (84.6) | 1302 (83.4) | 0.380 |  |
| Myalgia | 856 (54.8) | 789 (50.5) | 0.018 |  |
| Lymphadenectasis | 760 (48.7) | 730 (46.7) | 0.299 |  |
| Respiratory symptoms |  |  |  |  |
| Cough | | 683 (43.7) | 742 (47.5) | **0.037** |
| Sputum | | 505 (32.3) | 565 (36.2) | **0.026** |
| Dyspnea | | 70 (4.5) | 126 (8.1) | **<0.001** |
| Gastrointestinal symptoms | |  |  |  |
| Anorexia | 1262 (80.8) | 1285 (82.3) | 0.310 |  |
| Nausea | 1005 (64.3) | 980 (62.7) | 0.372 |  |
| Vomit | 553 (35.4) | 547 (35.0) | 0.851 |  |
| Abdominal | 180 (11.5) | 158 (10.1) | 0.226 |  |
| Diarrhea  Hemorrhagic signs | | 433 (27.7) | 391 (25.0) | 0.096 |
| Ecchymosis at  venipuncture site | 238 (15.2) | 267 (17.1) | 0.174 |  |
| Petechial | | 65 (4.2) | 56 (3.6) | 0.458 |
| Epistaxis | 15 (1.0) | 28 (1.8) | 0.065 |  |
| Gingival bleeding | 111 (7.1) | 91 (5.8) | 0.167 |  |
| Melena | 84 (5.4) | 83 (5.3) | 1.000 |  |
| Macroscopic hematuria | | 33 (2.1) | 48 (3.1) | 0.115 |
| Hemoptysis | 43 (2.8) | 37 (2.4) | 0.571 |  |
| Hematemesis | 20 (1.3) | 27 (1.7) | 0.378 |  |
| Neurological symptoms |  |  |  |  |
| Convulsion | 89 (5.7) | 128 (8.2) | **0.007** |  |
| Confusion | 188 (12.0) | 328 (21.0) | **<0.001** |  |
| Lethargy | 26 (1.7) | 58 (3.7) | **0.001** |  |
| Coma | 75 (4.8) | 170 (10.9) | **<0.001** |  |
| Convulsion | 210 (13.4) | 320 (20.5) | **<0.001** |  |

Continuous variables with skewed distribution were summarized as median and interquartile range (IQR). Categorical variables were summarized as frequency and proportion. *p* values were calculated by Wilcoxon rank sum tests and Chi-square tests where appropriate to estimate the differences between groups.

**Supplementary Table 2. Comparisons of demographic features and clinical manifestations between DM patients and non-DM patients.**

|  | Without DM (n=990) | With DM (n=495) | *P* |  |
| --- | --- | --- | --- | --- |
| Age, years | 67 (60, 72) | 66 (60, 72) | 0.866 |  |
| Male | 322 (32.5) | 165 (33.3) | 0.799 |  |
| Delay from symptom onset to admission, days | 6 (4, 7) | 6 (4, 7) | 0.810 |  |
| Hypertension | 399 (40.3) | 202 (40.8) | 0.896 |  |
| Non-specific features |  |  |  |  |
| Fever | 990 (100) | 495 (100) | - |  |
| Chills | 266 (26.9) | 135 (27.3) | 0.918 |  |
| Headache | 147 (14.8) | 67 (13.5) | 0.548 |  |
| Dizzy | 225 (22.7) | 112 (22.6) | 1.000 |  |
| Feeble | 846 (85.5) | 416 (84.0) | 0.521 |  |
| Myalgia | 535 (54.0) | 245 (49.5) | 0.110 |  |
| Lymphadenectasis | 504 (50.9) | 245 (49.5) | 0.646 |  |
| Respiratory symptoms |  |  |  |  |
| Cough | 436 (44.0) | 251 (50.7) | **0.018** |  |
| Sputum | 343 (34.6) | 191 (38.6) | 0.152 |  |
| Dyspnea | 59 (6.0) | 46 (9.3) | **0.024** |  |
| Gastrointestinal symptoms |  |  |  |  |
| Anorexia | 812 (82.0) | 406 (82.0) | 1.000 |  |
| Nausea | 692 (69.9) | 291 (58.8) | <0.001 |  |
| Vomit | 407 (41.1) | 163 (32.9) | 0.003 |  |
| Abdominal | 130 (13.1) | 53 (10.7) | 0.209 |  |
| Diarrhea | 302 (30.5) | 130 (26.3) | 0.102 |  |
| Hemorrhagic signs | |  |  |  |
| Ecchymosis at  venipuncture site | | 162 (16.7) | 78 (16.5) | 0.980 |
| Petechial | 73 (7.4) | 33 (6.7) | 0.695 |  |
| Epistaxis | 15 (1.5) | 5 (1.0) | 0.577 |  |
| Gingival bleeding | | 74 (7.5) | 32 (6.5) | 0.545 |
| Melena | 56 (5.7) | 24 (4.8) | 0.597 |  |
| Macroscopic hematuria | 22 (2.3) | 17 (3.6) | 0.199 |  |
| Hemoptysis | 23 (2.3) | 16 (3.2) | 0.389 |  |
| Hematemesis | 19 (1.9) | 9 (1.8) | 1.000 |  |
| Neurological symptoms |  |  |  |  |
| Convulsion | 71 (7.2) | 41 (8.3) | 0.509 |  |
| Confusion | | 157 (15.9) | 112 (22.6) | **0.002** |
| Lethargy | 26 (2.6) | 19 (3.8) | 0.261 |  |
| Coma | 75 (7.6) | 58 (11.7) | **0.011** |  |
| Convulsion | 158 (16.3) | 105 (22.2) | **0.008** |  |

Continuous variables with skewed distribution were summarized as median and interquartile range (IQR). Categorical variables were summarized as frequency and proportion. *p* values were calculated by Wilcoxon rank sum tests and Chi-square tests where appropriate to estimate the differences between groups.

**Supplementary Table 3. Comparisons of laboratory test results that were obtained at admission in patients with diabetes with those patients without diabetes.**

|  | Without DM (n=990) | With DM (n=495) | *p* |
| --- | --- | --- | --- |
| White blood cell count, ×10^9^/L | 3.05 (2.15, 4.64) | 3.10 (2.20, 4.90) | 0.243 |
| Lymphocyte percentage, % | 22.85 (15.83, 32.80) | 21.60 (15.50, 30.60) | 0.109 |
| Platelet count, ×10^9^/L | 56.00 (41.50, 78.00) | 57.00 (40.00, 74.00) | 0.271 |
| Neutrophil percentage, % | 69.97 (56.29, 78.33) | 70.72 (59.04, 78.61) | 0.304 |
| Albumin, g/L | 34.70 (31.33, 38.33) | 33.33 (30.09, 36.91) | <0.001 |
| Alanine transarninase, U/L | 55.50 (33.00, 107.50) | 58.05 (38.50, 100.62) | 0.193 |
| Aspartate aminotransferase, U/L | 117.00 (61.40, 248.30) | 117.25 (66.25, 228.88) | 0.878 |
| Lactate dehydrogenase, U/L | 505.7 (329.0, 808.9) | 563.0 (351.5, 873.5) | 0.033 |
| Blood urea nitrogen, mmol/L | 5.76 (4.20, 7.62) | 6.02 (4.54, 8.14) | 0.015 |
| Creatine kinase, U/L | 345.0 (155.9, 759.8) | 333.0 (137.0, 759.0) | 0.363 |
| Viral load, log_10_ copies/mL | 6.04 (5.24, 6.80) | 6.26 (5.59, 7.06) | 0.007 |

Continuous variables with skewed distribution were summarized as median and interquartile range (IQR). *p* values were calculated by Wilcoxon rank sum test to estimate the differences between groups.

**Supplementary Table 4. Comparisons of demographic features and clinical and laboratory characteristics at admission between patients receiving combined metformin and insulin treatment and patients receiving insulin alone.**

|  | With insulin treatment alone (n=180) | With combined metformin and insulin treatment (n=90) | *p* |  |
| --- | --- | --- | --- | --- |
| Age, years | 66 (60, 70) | 65 (58, 71) | 0.953 |  |
| Male | 64 (35.6) | 30 (33.3) | 0.821 |  |
| Delay from symptom onset to admission, days | 5 (4, 7) | 6 (4, 7) | 0.887 |  |
| Hypertension | 73 (40.6) | 37 (41.1) | 0.948 |  |
| Non-specific features |  |  |  |  |
| Fever | 180 (100) | 90 (100) | - |  |
| Chills | 39 (21.7) | 26 (28.9) | 0.247 |  |
| Headache | 21 (11.7) | 13 (14.4) | 0.650 |  |
| Dizzy | 32 (17.8) | 19 (21.1) | 0.621 |  |
| Feeble | 141 (78.3) | 67 (74.4) | 0.574 |  |
| Myalgia | 79 (43.9) | 30 (33.3) | 0.125 |  |
| Lymphadenectasis | 73 (40.6) | 31 (34.4) | 0.401 |  |
| Respiratory symptoms |  |  |  |  |
| Cough | 34 (18.9) | 26 (28.9) | 0.912 |  |
| Sputum | 34 (18.9) | 26 (28.9) | 0.088 |  |
| Dyspnea | 1 (0.6) | 1 (1.1) | 1.000 |  |
| Gastrointestinal symptoms |  |  |  |  |
| Anorexia | 121 (67.2) | 67 (74.4) | 0.282 |  |
| Nausea | 83 (46.1) | 43 (47.8) | 0.897 |  |
| Vomit | 47 (26.1) | 21 (23.3) | 0.729 |  |
| Abdominal | 5 (2.8) | 5 (5.6) | 0.425 |  |
| Diarrhea | 33 (18.3) | 17 (18.9) | 1.000 |  |
| Hemorrhagic signs |  |  |  |  |
| Ecchymosis at venipuncture  site | | 16 (8.9) | 7 (7.8) | 0.939 |
| Petechial | 5 (2.8) | 2 (2.2) | 1.000 |  |
| Gingival bleeding | | 4 (2.2) | 2 (2.2) | 1.000 |
| Melena | 2 (1.1) | 0 (0) | 1.000 |  |
| Macroscopic hematuria | 3 (1.7) | 0 (0) | 0.538 |  |
| Hematemesis | 1 (0.6) | 1 (1.1) | 1.000 |  |
| Neurological symptoms |  |  |  |  |
| Convulsion | 4 (2.2) | 2 (2.2) | 1.000 |  |
| Confusion | | 16 (8.9) | 2 (2.2) | 0.070 |
| Lethargy | 3 (1.7) | 0 (0) | 0.538 |  |
| Coma | 5 (2.8) | 1 (1.1) | 0.661 |  |
| Convulsion | 29 (16.1) | 8 (8.9) | 0.150 |  |
| Viral load, log_10_ copies/mL | 6.42 (5.79, 7.06) | 6.18 (5.55, 6.80) | 0.197 |  |
| White blood cell count, ×10^9^/L | 3.00 (2.20, 4.70) | 2.73 (1.90, 4.58) | 0.342 |  |
| Lymphocyte percentage, % | 21.60 (17.30, 28.95) | 21.48 (15.57, 30.33) | 0.658 |  |
| Platelet count, ×10^9^/L | 50.00 (39.38, 67.62) | 55.00 (41.12, 72.75) | 0.251 |  |
| Alanine transarninase, U/L | 57.50 (41.62, 110.12) | 61.00 (43.50, 89.00) | 0.583 |  |
| Lactate dehydrogenase, U/L | 485.0 (340.3, 813.7) | 431.5 (313.5, 672.2) | 0.387 |  |

Continuous variables with skewed distribution were summarized as median and interquartile range (IQR). Categorical variables were summarized as frequency and proportion. *p* values were calculated by Wilcoxon rank sum tests and Chi-square tests where appropriate to estimate the differences between groups.

**Supplementary Table 5. Oligonucleotides used in this study**

| **Gene** | **Primer Sequence** | **Reference** |
| --- | --- | --- |
| SFTSV-L | Forward: 5’- CTCACTCATGCCCTCAACGA -3’ | PMID: 34818556 |
|  | Reverse: 5’-GATGAACTCACCAGCCCTGC-3’ |  |
| Actin-hum | Forward: 5’-GCGGGAAATCGTGCGTGAC-3’ | PMID: 29883070 |
|  | Reverse: 5’-GGAAGGAAGGCTGGAAGAG-3’ |  |
| G6PC1-hum | Forward: 5’-TAAGTGGATTCTCTTTGGACAGC-3’ | PMID: 37186819 |
|  | Reverse: 5’- TTCCCTGGTCCAGTCTCACA-3’ |  |
| PCK2-hum | Forward: 5’-GGCTGAGAATACTGCCACACT-3’ | PMID: 37565737 |
|  | Reverse: 5’- ACCGTCTTGCTCTCTACTCGT-3’ |  |
| PC-hum | Forward: 5’-GCTAAACAGGTGGGCTACGA-3’ | PMID: 37186819 |
|  | Reverse: 5’- CGTCGGTGATCTCCTCTGTG-3’ |  |
| Glut2-hum | Forward: 5’- TTGGTGGGTGGCTTGGGGAC-3’ | PMID: 34035238 |
|  | Reverse: 5’- ACCAGGCCTGAAATTAGCCCACA-3’ |  |
| PFKL-hum | Forward: 5’- GGCATTTATGTGGGTGCCAAAGTC-3’ | PMID: 29038421 |
|  | Reverse: 5’- CAGTTGGCCTGCTTGATGTTCTCA-3’ |  |
| TPI1-hum | Forward: 5’-CCCAGGAAGTACACGAGAAG-3’ | PMID: 35509067 |
|  | Reverse: 5’- CAGTCACAGAGCCTCCATAAA-3’ |  |
| IFNB-hum | Forward: 5’-TCCAAATTGCTCTCCTGTTG-3’ | PMID: 31155231 |
|  | Reverse: 5’-GCAGTATTCAAGCCTCCCAT-3’ |  |
| ISG15-hum | Forward: 5’-CGCAGATCACCCAGAAGATCG-3’ | PMID: 34637813 |
|  | Reverse: 5’- TTCGTCGCATTTGTCCACCA-3’ |  |
| Actin-mus | Forward: 5’- GGAGATTACTGCCCTGGCTCCTA -3’ | PMID: 33318468 |
|  | Reverse: 5’- GACTCATCGTACTCCTGCTTGCTG -3’ |  |
| SFTSV-S | Forward: 5’- TTCACAGCAGCATGGAGAGG -3’ | PMID: 23965284 |
|  | Reverse: 5’- GATGCCTTCACCAAGACTATCAATG -3’ |  |
|  | Probe: 5’- AACTTCTGTCTTGCTGGTCCGC-3’ |  |

**Supplementary Table 6. Antibodies used in this study.**

| **Antibodies** | **Dilution** | **Source** | **Identifier** |
| --- | --- | --- | --- |
| Anti-GAPDH Mouse Monoclonal Antibody | 1:5000 | Applygen | Cat# C1312 |
| Beta Actin Recombinant antibody | 1:5000 | Proteintech | Cat# 81115-1-RR |
| phospho-Thr172-AMPK | 1:1000 | Cell Signaling Technology | Cat# 2535 |
| AMPK | 1:1000 | Cell Signaling Technology | Cat# 2532 |
| LC3B | 1:1000 | Cell Signaling Technology | Cat# 3868 |
| LC3B | 1:1000 | Cell Signaling Technology | Cat#2775 |
| Phospho-mTOR(Ser2448)(D9C2) | 1:1000 | Cell Signaling Technology | Cat# 5536 |
| mTOR (7C10) | 1:1000 | Cell Signaling Technology | Cat# 2983 |
| SQSTM1/P62 | 1:1000 | Cell Signaling Technology | Cat# 5114 |
| Peroxidase-conjugated Affnipure Goat Anti-Mouse IgG(H+L) | 1:10000 | Applygen | Cat# C1308 |
| Peroxidase-conjugated Affnipure Goat Anti-Rabbit IgG(H+L) | 1:10000 | Applygen | Cat# C1309 |
| Rabbit polyclonal anti-NP | 1:1000 | This paper | N/A |
| Mouse polyclonal anti-NP | 1:1000 | This paper | N/A |
| fluorescein-5-isothiocyanate–conjugated goat anti-mouse IgG | 1:1000 | TransGen Biotech | Cat#HS211-01 |
| anti-IFNAR1 IgG | - | Bio X Cell | Cat# BE0241 |
